# Cell and molecular profiles in peripheral nerves shift toward inflammatory phenotypes in diabetic peripheral neuropathy

**DOI:** 10.1172/JCI184075

**Published:** 2025-08-19

**Authors:** Diana Tavares-Ferreira, Breanna Q. Shen, Juliet M. Mwirigi, Stephanie Shiers, Ishwarya Sankaranarayanan, Akshitha Sreerangapuri, Miriam B. Kotamarti, Nikhil N. Inturi, Khadijah Mazhar, Eroboghene E. Ubogu, Geneva L. Thomas, Trapper Lalli, Shai M. Rozen, Dane K. Wukich, Theodore J. Price

**Affiliations:** 1 Department of Neuroscience and Center for Advanced Pain Studies, University of Texas at Dallas, Richardson, Texas, USA.; 2 Department of Neurology, Division of Neuromuscular Disease, University of Alabama at Birmingham, Birmingham, Alabama, USA.; 3 Department of Orthopedic Surgery and; 4 Department of Plastic Surgery, UT Southwestern Medical Center, Dallas, Texas, USA.

**Keywords:** Inflammation, Neuroscience, Diabetes, Neurodegeneration, Pain

## Abstract

Diabetic peripheral neuropathy (DPN) is a prevalent complication of diabetes mellitus caused by metabolic toxicity to peripheral axons. We aimed to gain deep mechanistic insight into the disease using transcriptomics on tibial and sural nerves recovered from lower leg amputations in a mostly diabetic population and control sural nerves from cross-facial nerve graft surgery. First, comparing DPN versus control sural nerves revealed inflammatory activation and sensory changes in DPN. Second, when comparing mixed sensory and motor tibial and purely sensory sural nerves, we identified key pathway differences in affected DPN nerves, with distinct immunological features observed in sural nerves. Third, spatial transcriptomics of sural nerves revealed shifts in immune cell types associated with axonal loss progression. We also found clear evidence of neuronal transcript changes, like *PRPH*, in nerves with axonal loss, suggesting perturbed RNA transport into distal sensory axons. This motivated further investigation into neuronal mRNA localization in peripheral nerve axons, generating evidence of robust localization of mRNAs such as *SCN9A* and *TRPV1* in human sensory axons. Our work provides insight into altered cellular and transcriptomic profiles in human nerves in DPN and highlights sensory axon mRNA transport as a potential contributor to nerve degeneration.

## Abstract 2

Transcriptomic analysis of unique human control and DPN nerves reveals inflammatory, vascular, and neuronal changes, highlighting novel mechanisms underlying DPN progression.

## Introduction

Diabetes mellitus is a public health problem, and diabetic peripheral neuropathy (DPN) is one of its most common and debilitating consequences, affecting an estimated 37.3 million people in the United States alone (1, 2) and 463 million people worldwide (3, 4). Distal symmetric sensory polyneuropathy is the most common form of DPN, accounting for approximately 75% of DPN cases (4). The prevalence of DPN is estimated to be at least 20% in patients with type 1 diabetes after 20 years and 50% in patients with type 2 diabetes after 10 years (4).

DPN affects peripheral sensory nerves, and the long axons that innervate the feet are usually the first to be affected. DPN causes pain in about half of affected individuals, making it the most common cause of neuropathic pain (5). Many transcriptomic, proteomic, and lipidomic studies have been published using DPN rodent models to understand the underlying pathology in peripheral nerves that causes neurodegeneration and pain in diabetes (6–13). In rodent models there is clear evidence for inflammation driven by endoneurial immune cell infiltration causing increased oxidative stress and neuroimmune signaling that results in nociception and promotes axonal degeneration (7, 9, 10). Accompanying changes in the proteome (13) and lipid profile (6, 14) of peripheral nerves in rodent diabetes models are consistent with transcriptomic studies. We are unaware of any previous studies examining transcriptomic profiles of human peripheral nerves from DPN patients; however, transcriptomic, proteomic, and metabolomic studies have been conducted on human dorsal root ganglia (DRG) recovered from organ donors who died with a history of DPN pain (15–17). These studies highlight signs of neurodegeneration (15, 17), macrophage proliferation or infiltration (15), and altered mRNA processing (16) within the DRG associated with pain in DPN. A primary goal of our work was to use RNA sequencing to compare control and DPN sural nerves and to uncover molecular changes in both tibial and sural nerves associated with DPN. Our underlying hypothesis was that this approach would reveal molecular insight into the extensive neurodegeneration and accompanying immune cell infiltration observed in animal studies and in previous human pathology studies.

Human DRG neurons that give rise to sensory axons have a highly polarized structure with axons that extend a large distance away from the nucleus and cell body (18, 19). Measurements from a study examining peripheral nerves in human cadavers show that the average length of the tibial nerve was 74.3 cm and that of the sural nerve was 38.4 cm (20), both of which were measured after origination from the sciatic nerve, making these nerves over a meter in length in most humans. The long length of these nerves presents a challenge for maintaining the integrity of the axonal proteome, because axonal transport rates from the soma do not readily account for rapid changes in protein content that can occur in axons, and these axonal transport rates are also inconsistent with maintenance of basic functions in very long axons, such as those that innervate the extremities in humans (19, 21–23). To overcome this problem, peripheral axons transport mRNAs that can be translated locally to rapidly respond to external signals, and to maintain the functional integrity of axons (24). The majority of studies examining RNA transport into DRG axons have been done using rodent DRG neurons in primary cultures (25–28), although there is also strong support from in vivo studies for RNA transport and local translation of mRNA in DRG axons in rodents (23, 29–35). No studies have been done to assess axonal mRNAs in human peripheral axons, although we have recently demonstrated accumulation of the RNA-binding protein (RBP) FMRP in the central terminals of human DRG neurons (36). The secondary goal of our study was to ascertain which human peripheral nerve mRNAs originate from sensory axons using a combination of multiple transcriptomic resources, followed by confirmation by RNA in situ hybridization. This characterization lays the groundwork for studying how altered axonal mRNA transport and translation contribute to nerve dysfunction in DPN.

In this study we used sural and tibial nerves recovered from lower leg amputation surgeries and control sural nerves from cross-facial nerve graft to (a) compare control versus DPN sural nerves to define disease-associated transcriptional changes, (b) gain insight into DPN pathogenesis and identify differences between sensory and mixed peripheral nerves, and (c) begin to unravel how mRNA transport into human DRG axons may play a key role in maintaining axonal integrity in health and disease. Our findings give insight into advanced DPN pathogenesis, providing evidence for major reorganization of the Schwann cells and fibroblasts and alteration of endothelial and infiltrating immune cell signature with disease progression. Our work also demonstrates pervasive transport of neuronal mRNAs into sensory axons with evidence that this process is guided by specific RBPs that likely bind motifs in the 3′-UTRs (37–39) of these mRNAs to move them into distal axons.

## Results

### Gene expression profiling of DPN and control sural nerves reveals inflammatory activation and sensory changes in DPN.

**Figure 1:**
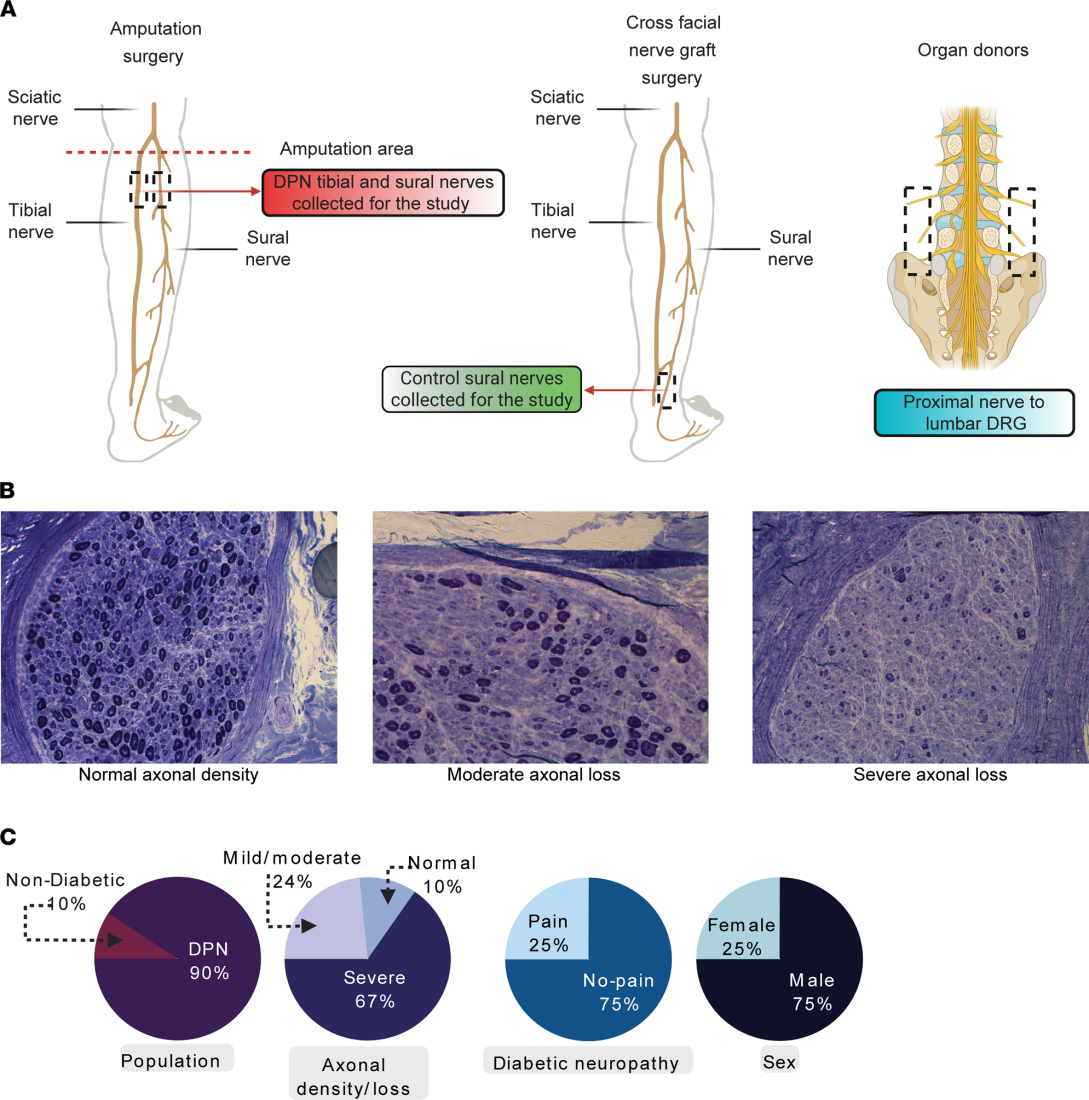
Nerve samples included in the study. (**A**) Tibial and sural nerves were harvested during lower-limb amputation procedures; control sural nerves were obtained during cross-facial nerve graft surgeries, and additional control peripheral nerves were harvested from organ donors. (**B**) Representative micrographs illustrating axonal density in sural nerves from patients with diabetic peripheral neuropathy (DPN) undergoing amputation. Original magnifications (left to right): ×200, ×400, and ×200. (**C**) Cohort and sample characteristics for the amputation group.

**Figure 2:**
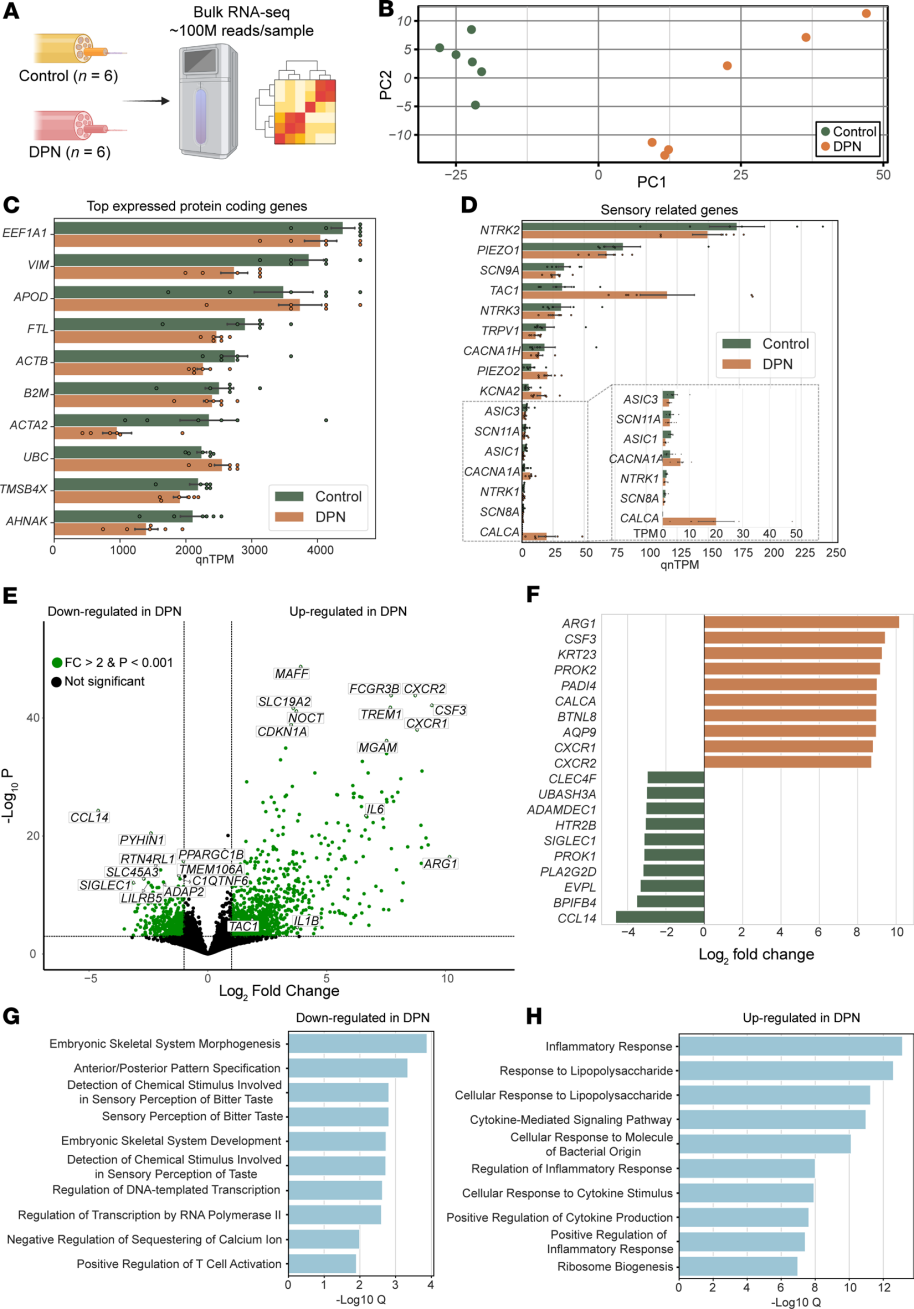
Transcriptomic profiling of human sural nerves reveals gene expression changes in DPN. (**A**) Bulk RNA sequencing was performed on sural nerve samples from control and DPN patients. (**B**) Principal component analysis shows clear separation between control and DPN samples. (**C**) Top expressed protein-coding genes in control and DPN samples. (**D**) Expression levels of sensory-related genes. (**E**) Volcano plot showing top differentially expressed genes and selected genes (*IL6*, *IL1B*, and *TAC1*) in DPN (fold change > 2, adjusted *P* value < 0.001). (**F**) Top 10 upregulated (orange) and downregulated (green) genes in DPN based on fold change. (**G** and **H**) Gene ontology analysis reveals enrichment among downregulated genes (**G**) and among upregulated genes (**H**) in DPN.

The study design, representative DPN sural nerve morphological photomicrographs, and the study population and sample characteristics are shown in Figure 1 and Supplemental Data File 1 (supplemental material available online with this article; https://doi.org/10.1172/JCI184075DS1). To investigate transcriptional changes associated with DPN, we performed bulk RNA sequencing on sural nerve samples from control donors (*n* = 6) and patients with DPN (*n* = 6) (Supplemental Table 1), generating about 100 million reads per sample (Figure 2A and Supplemental Data File 2). Principal component analysis revealed clear separation between control and DPN samples, indicating distinct transcriptomic profiles (Figure 2B). Among the top expressed protein-coding genes were *EEF1A1* and *VIM*, which are involved in fundamental cellular processes such as protein translation and cytoskeletal organization, respectively (Figure 2C). We also detected several sensory-related genes, including ion channels and neurotrophic receptors, expressed in both control and DPN sural nerves (Figure 2D). Differential gene expression analysis identified 2,268 genes that were significantly altered (fold change > 2, adjusted *P* value < 0.001), with proinflammatory mediators such as *CXCL2*, *CSF3*, *IL1R2*, *IL6*, and *IL1B* and neuropeptides *CALCA* and *TAC1* upregulated in DPN (Figure 2, E and F, and Supplemental Data File 3). Among the downregulated genes in DPN, *SIGLEC1* and *PROK1* stand out for their roles in immune regulation and neurovascular signaling, respectively. *PLA2G2D*, involved in antiinflammatory lipid metabolism, was also reduced, suggesting impaired resolution of inflammation in DPN nerves (Figure 2, E and F). Gene ontology analysis revealed downregulation of pathways involved in neurodevelopment and sensory signaling (Figure 2G and Supplemental Data File 4), whereas upregulated genes were enriched for inflammatory response, cytokine signaling, and innate immune activation (Figure 2H and Supplemental Data File 5), validating immune dysregulation as a key feature of DPN pathology (15, 40, 41).

### Sensory and mixed nerves show distinct molecular responses in DPN.

**Figure 3:**
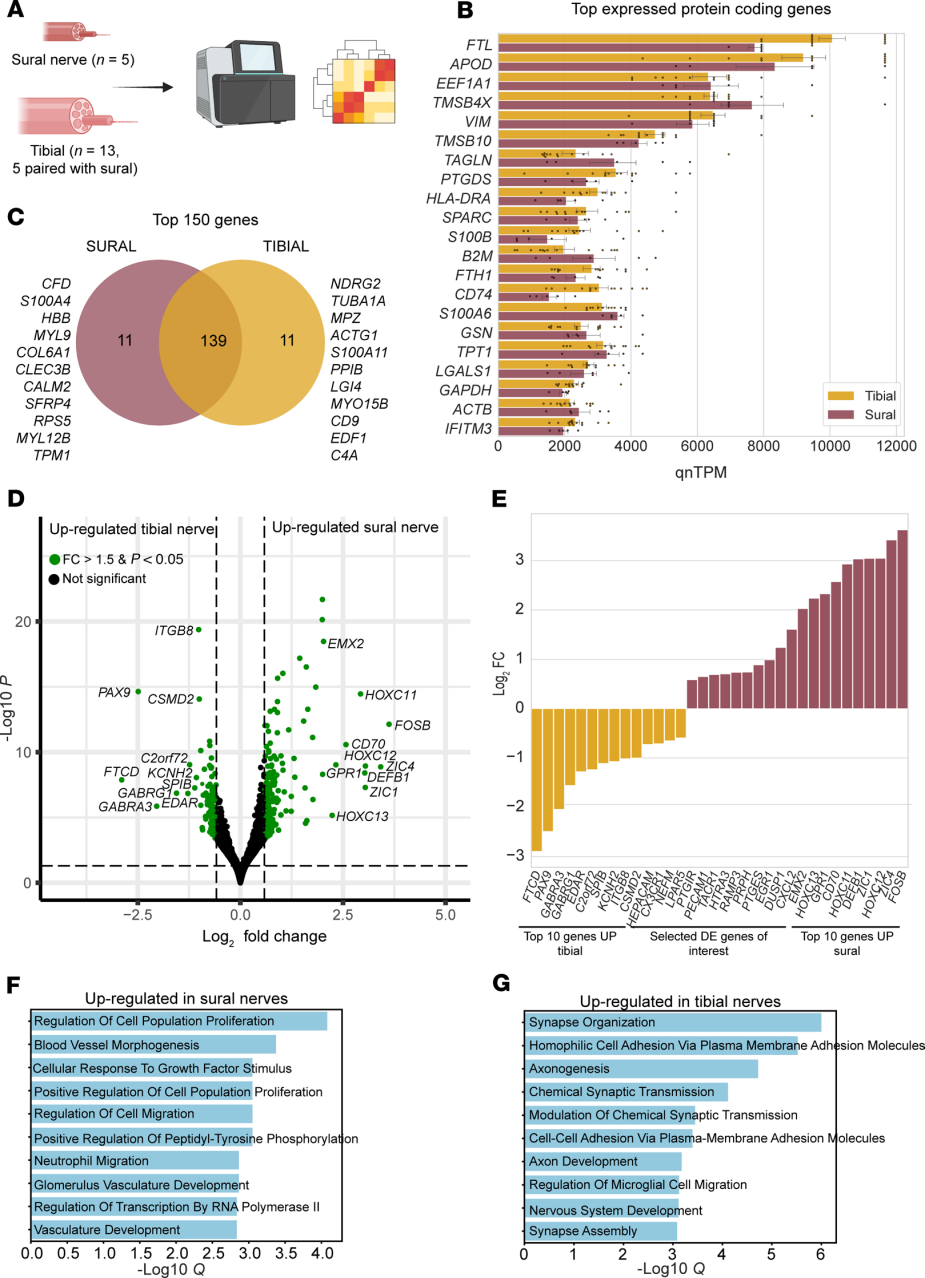
Comparisons between paired tibial and sural nerves. (**A**) Overview of samples used for analysis. (**B**) Top protein-coding genes (excluding ribosomal protein genes). (**C**) Overlap between sural and tibial top expressed genes. (**D**) Differences in gene expression between sural and tibial nerve (fold change > 1.5, adjusted *P* value < 0.05). (**E**) Top differentially expressed genes. (**F** and **G**) Top 10 *q* values for Gene Ontology biological process 2023 for genes upregulated in sural (**F**) and tibial (**G**) nerves. *Q* = adjusted *P* value.

Next, we sought to conduct a comprehensive analysis of the tibial and sural nerves (Supplemental Table 2) using bulk and spatial RNA sequencing and observed that the top expressed genes were similar in these nerves (Figure 3, A and B, and Supplemental Data File 6). Among the top expressed genes were ferritin light chain (*FTL*), involved in iron accumulation, and apolipoprotein D (*APOD*), a glia-derived apolipoprotein that has been implicated in maintaining peripheral nerve function (42). Because we had paired tibial and sural samples from the same patients (*N* = 5), we sought to examine distinct patterns in gene expression between the sural and tibial nerves. We identified a total of 321 differentially expressed genes between sural and tibial nerves (Figure 3C and Supplemental Data File 7). We observed that genes such as *FOSB*, *CXCL2*, *EGR1*, *PTGES*, and *PRPH* were upregulated in sural nerves, while *GABRA3*, *HEPACAM*, *CX3CR1*, *NEFM*, and *PTGIR* were upregulated in tibial nerves (Figure 3D). We performed gene enrichment analysis to uncover pathways associated with these differentially expressed genes and noted that genes upregulated in sural nerves were involved primarily in non-neuronal pathways, including blood vessel and vasculature development and neutrophil migration (Figure 3E and Supplemental Data File 8). In contrast, genes upregulated in tibial nerves were involved in axonogenesis, axon development, and glial cell migration (Figure 3F and Supplemental Data File 9). This suggests that there are molecular divergences between sensory and mixed peripheral nerves in tibial and sural nerves following DPN.

### Progressive cellular remodeling with axonal loss in DPN reflects a shift from perineurial disruption to immune infiltration and fibrosis.

**Figure 4:**
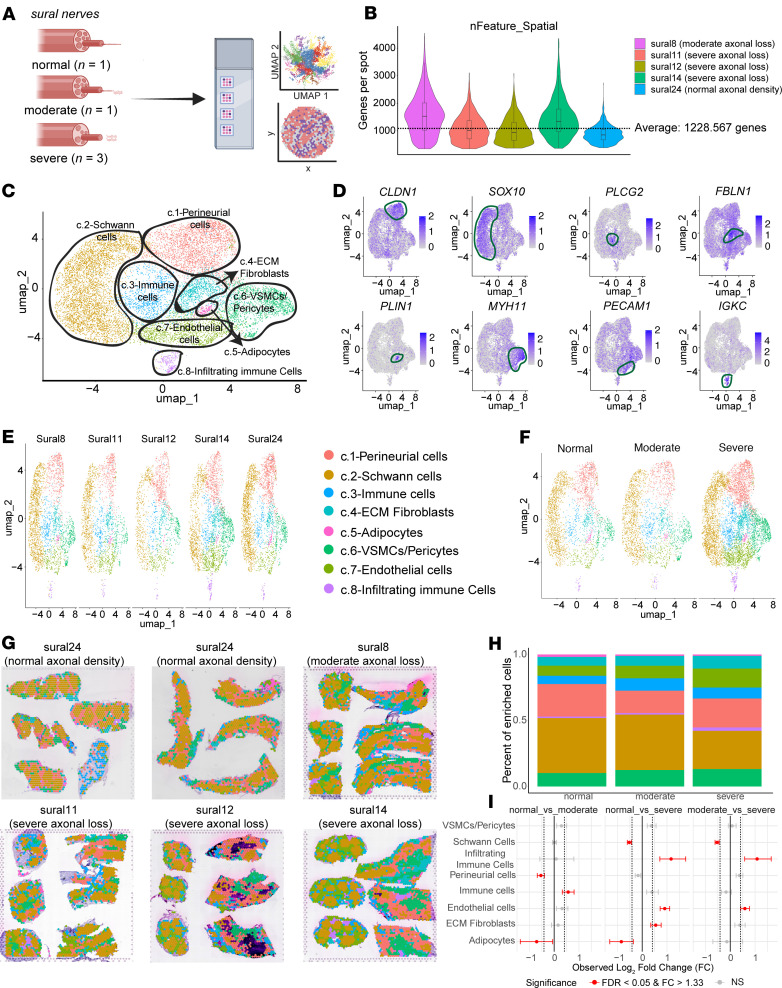
Spatial transcriptomic profiling of human sural nerves with varying degrees of axonal loss. (**A**) Overview of sample selection and processing. (**B**) Violin plot showing the distribution of detected genes per spot across all samples. Dotted line indicates the average gene count (1,228.6 genes per spot). (**C**) UMAP embedding of all transcriptomic spots, clustered into 8 major cell types. (**D**) Spatial expression and cluster-level localization of canonical marker genes for each respective cell type. (**E** and **F**) UMAP projections of cell type annotations across individual samples (**E**) and grouped by neuropathy severity (**F**). (**G**) Spatial projections showing cell type annotations overlaid on tissue sections for each sural nerve sample. Colors correspond to cell types identified in **C** and **E**. (**H**) Stacked-bar plot showing the proportion of each enriched cell type across samples stratified by axonal loss severity. (**I**) Differential abundance analysis of cell types between severity groups. Log_2_ FC values are shown for each pairwise comparison. Red indicates statistically significant changes (FDR < 0.05 and FC >1.33); gray indicates non-significant (n.s.) differences.

Next, we leveraged Visium spatial transcriptomics (10x Genomics), which maintains spatial context, to characterize sural nerves with different degrees of axonal loss (Figure 4 and Supplemental Figure 1). Visium spatial transcriptomics uses 55 μm barcoded spots printed on specialized slides. The diameter of myelinated axons in human sural nerves ranges from 9 to 12 μm in adults (43), Schwann cells have a length ranging from 220 to 400 μm (with their thickness ranging between 2 and 5 μm) (44), immune cells such as macrophages can be on average 21 μm (45), and T cells vary between 8 and 10 μm in diameter (46). Therefore, we first wanted to characterize the enriched cell types in a given barcoded spot. To characterize cellular composition across human sural nerves with varying degrees of axonal loss, we applied the Seurat layer integration workflow to 6 spatial transcriptomic datasets spanning normal, moderate, and severe axonal loss samples (Figure 4A). On average, about 1,229 genes per spot were detected across samples (Figure 4B). Unsupervised clustering and dimensionality reduction identified 8 major enriched cell types: perineurial cells (*CLDN1^+^*), Schwann cells (*SOX10^+^*), immune cells (*PLCG2^+^*), extracellular matrix (ECM) fibroblasts (*FBLN1^+^*), adipocytes (*PLIN1^+^*), vascular smooth muscle cells/pericytes (*MYH11^+^*), endothelial cells (*PECAM1^+^*), and infiltrating immune cells (*IGKC^+^*) (Figure 4, C and D). Cell type distributions were consistent across individual samples (Figure 4E) and axonal loss groups (Figure 4F). We next assessed how the enriched cell types were projected onto the tissue sections using spatial mapping. Spatial plots of individual samples showed consistent anatomical localization of major cell types across normal, moderate, and severe axonal loss samples but apparent differences in their proportions (Figure 4G). Quantification of enriched cell type proportions revealed a clear shift in tissue composition with increasing axonal loss, characterized by a reduction in Schwann cells and a corresponding increase in ECM fibroblasts, immune cells, and vascular/endothelial populations (Figure 4H). Statistical comparison using permutation-based testing identified significant changes (FDR < 0.05, fold change > 1.33) in several cell populations across stages of axonal loss (Figure 4I). Notably, perineurial cells were altered between normal and moderate samples, suggesting early disruption of the nerve barrier architecture. In the transition from moderate to severe axonal loss, we observed an increase in infiltrating immune cells and endothelial cells, consistent with progressive inflammation and vascular remodeling. ECM fibroblasts were changed only when normal samples were compared with severe, indicating that fibrotic remodeling may be a later event in disease progression. Schwann cells were consistently reduced in severe samples compared with both moderate and normal nerves. Collectively, these findings point to a staged pattern of cellular disruption in DPN, beginning with perineurial changes, followed by immune and vascular infiltration, and culminating in fibrotic expansion and Schwann cell loss in advanced disease.

Using RNAscope and immunohistochemistry (IHC) (2 different antibodies), we found that *SOX10* mRNA transcripts were highly colocalized with SOX10 protein (Supplemental Figure 2). Additionally, *SOX10* mRNA puncta were particularly localized in Schwann cells surrounding nerve fibers that colocalized with DAPI, which stains cell nuclei. Schwann cells, the glial cells in peripheral nerves, are key to maintaining axonal homeostasis, supporting them through release of neurotrophins such as nerve growth factor (NGF) (47), providing rationale to further evaluate how this cell type changed in nerves from DPN patients with moderate and severe axonal loss. Next, we sought to confirm our findings using an independent approach choosing cell type deconvolution to examine cell proportions within barcodes. We used a reference-free cell type deconvolution method, STdeconvolve (48), and characterized moderate and severe axonal loss DPN sural nerves (the Visium frame for these samples contained both transverse and longitudinal sections). We observed similar results to our enriched cell type analysis and demonstrated that cell types drastically change with axonal loss severity (Supplemental Figure 3). Using cell type deconvolution, we identified gene expression markers for axons, myelinating Schwann cells, smooth muscle cells, perineurium, and other connective layers in both nerves with moderate and severe axonal loss. Non-myelinating Schwann cells were present only with moderate axonal loss. Additionally, mitochondrial gene expression was observed in DPN with severe axonal loss, implying activation of oxidative stress pathways with advanced disease.

### Intercellular signaling analysis reveals pathways underlying the transition from regenerative to profibrotic states with progressive axonal loss in DPN.

**Figure 5:**
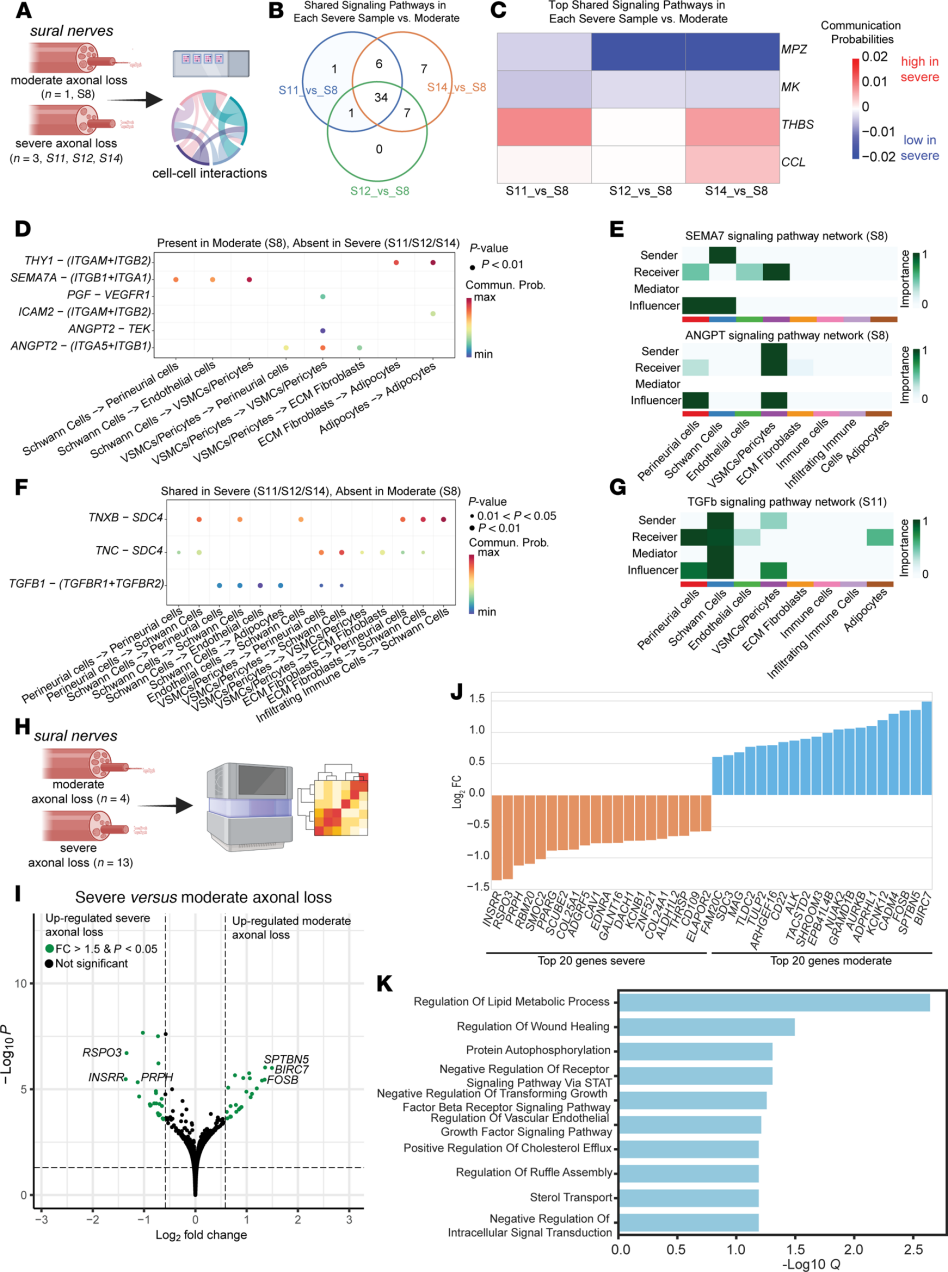
Comparative analysis in sural nerves with moderate versus severe axonal loss. (**A**) Overview of spatial transcriptomic samples used for cell-cell interaction analysis. (**B**) Venn diagram showing the number of signaling pathways shared between each severe sample and the moderate axonal loss sample. (**C**) Heatmap of communication probabilities for top shared signaling pathways across severe-versus-moderate comparisons. Red indicates pathways enriched in severe samples; blue indicates pathways enriched in moderate axonal loss sample. (**D**) Dot plot of ligand-receptor pairs and associated sender-receiver cell type pairs that are uniquely present in the moderate sample (S8). (**E**) Cell type–level communication roles for the SEMA7 and ANGPT signaling pathways in the moderate sample, showing sender, receiver, mediator, and influencer roles across cell types. (**F**) Dot plot of ligand-receptor pairs uniquely shared across severe samples. (**G**) Cell type–level communication roles in the TGF-β signaling network in a representative severe sample (S11). (**H**) Overview of moderate and severe axonal loss samples used for bulk RNA sequencing analysis. (**I**) Differential gene expression between sural nerves with severe versus moderate axonal loss (fold change > 1.5, adjusted *P* value < 0.05). (**J**) Top genes differentially expressed. (**K**) Gene enrichment analysis shows the top pathways affected by the differentially expressed genes. *Q* = adjusted *P* value.

Using CellChat (49), we compared sural nerves from 1 moderate (S8) and 3 severe (S11, S12, S14) DPN samples to assess changes in intercellular signaling (Figure 5A). This comparison aimed to identify how signaling networks shift with increasing axonal loss. We selected these samples because they had matched transverse and longitudinal sections, ensuring anatomical consistency. As detailed in Methods, we assessed both consistent signaling changes across individual comparisons (S8 vs. each severe sample) and pathways uniquely enriched in either moderate or severe axonal loss. Comparison of signaling networks revealed overlap in enriched pathways across severe axonal loss nerves relative to the moderate sample (Figure 5B). Among the pathways shared across all 3 severe versus moderate comparisons, MPZ, MK, THBS, and CCL emerged as consistently altered (Figure 5C). MPZ and MK signaling was reduced in severe nerves, potentially reflecting loss of axon-glia communication and impaired neurotrophic support. In contrast, THBS and CCL signaling was upregulated, indicating increased ECM remodeling and inflammatory chemokine activity. Their consistent alteration across the 3 severe samples suggests that these pathways represent core features of advanced nerve pathology. Several pathways were uniquely present in moderate axonal loss nerve and absent in all severe cases, including SEMA7, THY1, ANGPT2, ICAM2, and VEGF (Figure 5D). Centrality analysis confirmed that SEMA7 and ANGPT2 act as key modulators of Schwann cell and vascular-stromal communication, respectively, in the moderate sample (Figure 5E), highlighting a pro-regenerative signaling environment that remains present in moderate axonal loss but is absent with axonal loss progression. In contrast, nerves with severe axonal loss showed enrichment of profibrotic pathways including TGF-β (TGFb) and tenascin, which was absent in the moderate sample (Figure 5F). TGFb pathway involves coordinated signaling between perineurial cells, endothelial cells, and Schwann cells, suggesting activation of multicellular fibrosis and remodeling networks in severe axon loss pathology (Figure 5G). Together, these findings highlight a pathological transition from a reparative to a profibrotic signaling and immune-infiltrated environment with disease progression. We also analyzed possible interactions between ligands expressed by these enriched cell groups and receptors that are present in human DRG neurons (Supplemental Figure 4). We identified several interactions that are driven by ligands such as amyloid-β precursor protein (*APP*), which is involved in fiber organization and neuron remodeling. This suggests that the ligands expressed in sural nerves are likely involved in axonal degeneration and regeneration.

Guided by considerable changes in peripheral cell types associated with changes in axonal density, we compared the transcriptome of DPN sural nerves with moderate and severe axonal loss using bulk RNA sequencing data (Figure 5H, Supplemental Table 3, and Supplemental Data File 10). Nerves with moderate axonal loss were significantly enriched (fold change >1.5, adjusted *P* value < 0.05) in genes such as baculoviral IAP repeat containing 7 (*BIRC7*), which is a family member of inhibitors of apoptosis (50), ALK receptor tyrosine kinase (*ALK*), and myelin-associated glycoprotein (*MAG*). Nerves with severe axonal loss showed an increase in genes such as peripherin (*PRPH*), caveolin-1 (*CAV1*), and collagen type XXV alpha 1 chain (*COL25A1*) (Figure 5, I and J). Using imaging-based spatial transcriptomics (Xenium, 10x Genomics) and its predesigned brain panel, we verified that *ALK* is expressed mostly in immune cells, and *MAG* is expressed in Schwann cells. *COL25A1* and *CAV1* are expressed in fibroblasts; *CAV1* is also expressed in endothelial cells (Supplemental Figure 5). These data suggest that multiple peripheral cell types (immune cells, fibroblasts, Schwann cells, and endothelial cells) are altered and involved in the pathogenesis of advanced DPN with moderate and several axonal loss. Our gene enrichment analysis showed that differentially expressed genes increased in severe axonal loss were involved in metabolic process, wound healing, and signaling pathways such as signal transducer and activator of transcription (STAT) and transforming growth factor-β (TGFB) receptor pathways (Figure 5K and Supplemental Data File 11). These results are consistent with our findings from Visium spatial transcriptomics analysis and further support the involvement of multicellular profibrotic and inflammatory programs in progressive DPN.

### Specific mRNAs are localized to the axon of human peripheral nerves.

**Figure 6:**
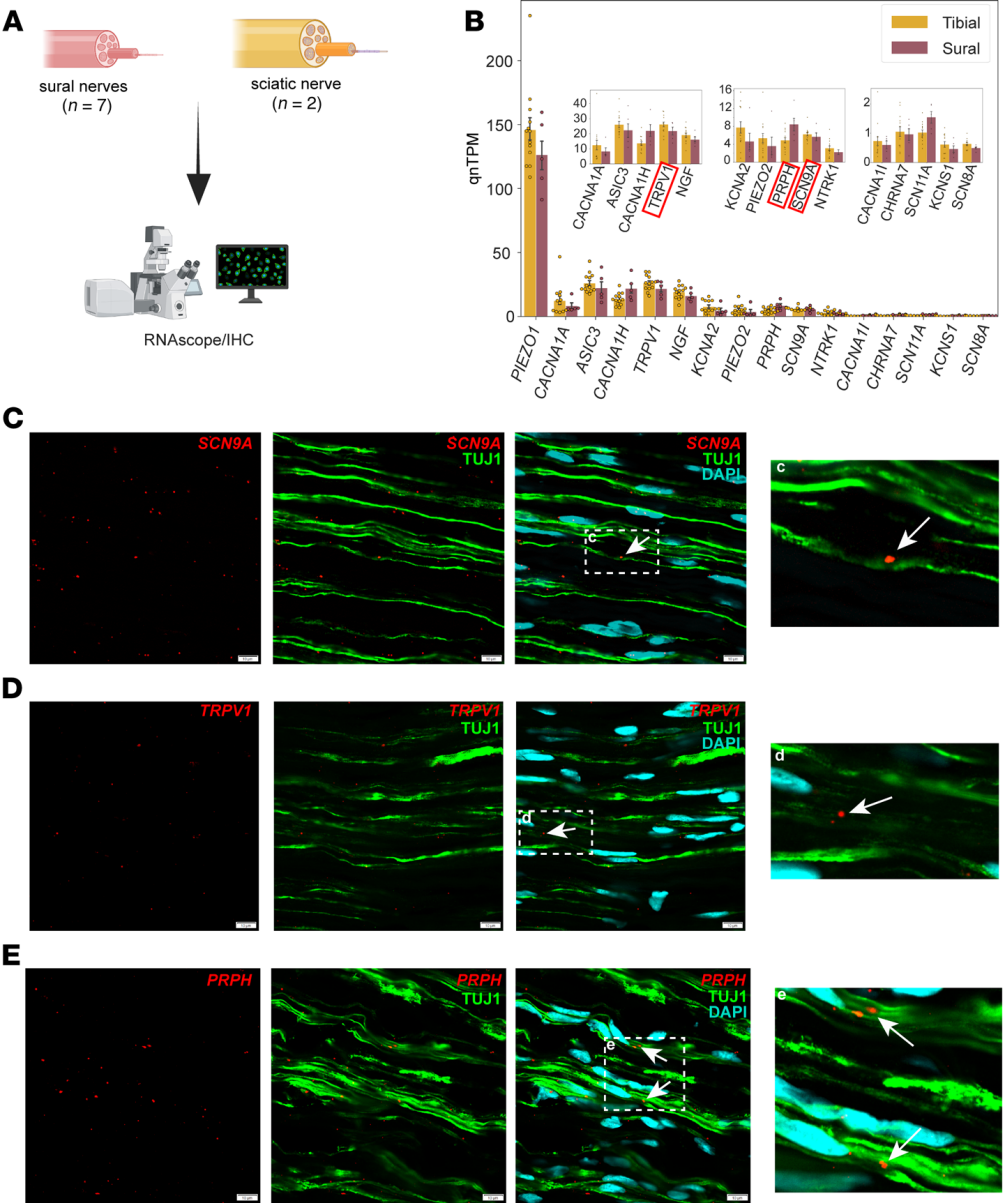
Gene expression in human peripheral nerves. (**A**) Overview of samples used for RNAscope/IHC. (**B**) We performed bulk RNA sequencing of human tibial and sural nerves (from Figure 3) and detected the presence of several mRNAs involved in sensory processing, such as *TRPV1* and *SCN9A*. (**C**–**E**) mRNA puncta of *SCN9A* (**C**), *TRPV1* (**D**), and *PRPH* (**E**) (red) are colocalized with β-tubulin III (TUJ1; green), which labels nerve fibers. Arrows point to areas where mRNA puncta do not overlap with DAPI (cyan), suggesting that it is axon-specific staining. Insets show zoomed-in images (approximate enlargement factors: ×3.5 [**C**]; ×3.2 [**D**]; ×2.9 [**E**]). Scale bars: 10 μm.

The differences we identified at the mRNA level between sural nerves with moderate and severe axonal loss led us to investigate axonal mRNA localization. This is an unstudied area of human sensory neuron research; however, previous rodent studies have shown that local translation is crucial for the maintenance of axonal homeostasis and regeneration (27, 51, 52). As axonal degeneration is commonly observed in peripheral neuropathies, including DPN, we examined the expression of neuronal markers in human peripheral nerves (Figure 6A). Using bulk RNA sequencing experiments, we detected neuronal markers such as transient receptor potential vanilloid 1 (*TRPV1*) and sodium voltage-gated channel α subunit 9 (*SCN9A*) in human tibial and sural nerves (Figure 6B). We also conducted a comprehensive meta-analysis of previously published studies (Supplemental Figure 6A) and identified a distinct set of genes that appear to be exclusively in sensory axons, representing putative axonal mRNAs (Supplemental Figure 6B). To strengthen our hypothesis that these genes are axonal and originate from the soma, we show their expression in human DRG neurons (Supplemental Figure 6C). Next, we performed RNAscope in situ hybridization on human sural and sciatic nerves for the neuronal markers *SCN9A*, *TRPV1*, *PRPH*, neurotrophic receptor tyrosine kinase 1 (*NTRK1*), and sodium voltage-gated channel α subunit 10 (*SCN10A*) followed by IHC to label cell nuclei (DAPI) and axons using β-tubulin III/TUJ1 (Figure 6, C–E) and peripherin (Supplemental Figures 7 and 8) to provide direct evidence for RNA localization for these genes in human peripheral nerve axons. While peripherin, an intermediate filament important for neuronal function, has been shown to change with axonal damage, β-tubulin III, a pan-neuronal marker, has not been reported to change in DPN models (53–55).

*SCN9A*, which encodes Na_v_1.7, colocalized with β-tubulin III (TUJ1) and peripherin in sural and sciatic nerves (Figure 6C, Supplemental Figure 7A, and Supplemental Figure 8A). Overlap of *SCN9A* puncta with peripherin was present even in areas where there was no DAPI staining, suggesting that these mRNAs exist both in the nuclei of axon-supporting cells and in the nerve axons. While we did not quantify RNA expression levels, the number of *SCN9A* puncta was comparable between sural and sciatic nerves. Colocalization with β-tubulin III or peripherin, in areas without DAPI staining, was also seen in sural and sciatic nerves for mRNA puncta of *TRPV1* (Figure 6D, Supplemental Figure 7B, and Supplemental Figure 8B), which encodes the transient receptor potential vanilloid 1 nonselective cation channel receptor, and *PRPH* (Figure 6E, Supplemental Figure 7C, and Supplemental Figure 8C). *NTRK1* (Supplemental Figure 7D and Supplemental Figure 8D), which encodes the TrkA receptor tyrosine kinase, also colocalized with peripherin staining. While *SCN10A* (which encodes Na_v_1.8) was not detected with tibial and sural nerve bulk RNA sequencing, Na_v_1.8 has an important role in nociception, so we decided to further investigate *SCN10A* axonal expression with a more sensitive technique, guided by previous reports of this mRNA localizing to rat sensory axons originating from DRG (29, 56). We observed only a few mRNA puncta colocalized with peripherin; however, this observation suggests transport for this mRNA in human peripheral nerve axons as well (Supplemental Figure 7E and Supplemental Figure 8E). The difference in sensitivity between bulk RNA sequencing and RNAscope may contribute to the detection of a few *SCN10A* transcripts with the latter approach. In line with our bulk RNA sequencing data, our qualitative assessment of mRNA puncta suggests that *SCN9A* is more highly expressed in sural and sciatic nerves than the other mRNAs. Additionally, using a high-resolution imaging–based Xenium approach, we were able to detect neuronal markers such as parvalbumin (*PVALB*), tachykinin precursor 1 (*TAC1*), and transient receptor potential cation channel subfamily C member 5 (*TRPC5*) in peripherin-labeled human peripheral axons in longitudinal and transverse sections (Supplemental Figure 9). We also examined markers of neuronal subtypes previously identified in the human DRG (57), and we identified mRNA expression for 96 of 126 unique gene markers (76.19%) in the human tibial and sural nerve bulk RNA sequencing data (Supplemental Figure 10). Identification of specific neuronal subpopulation markers can provide important information for the identification of specific subtypes potentially affected in DPN.

**Figure 7:**
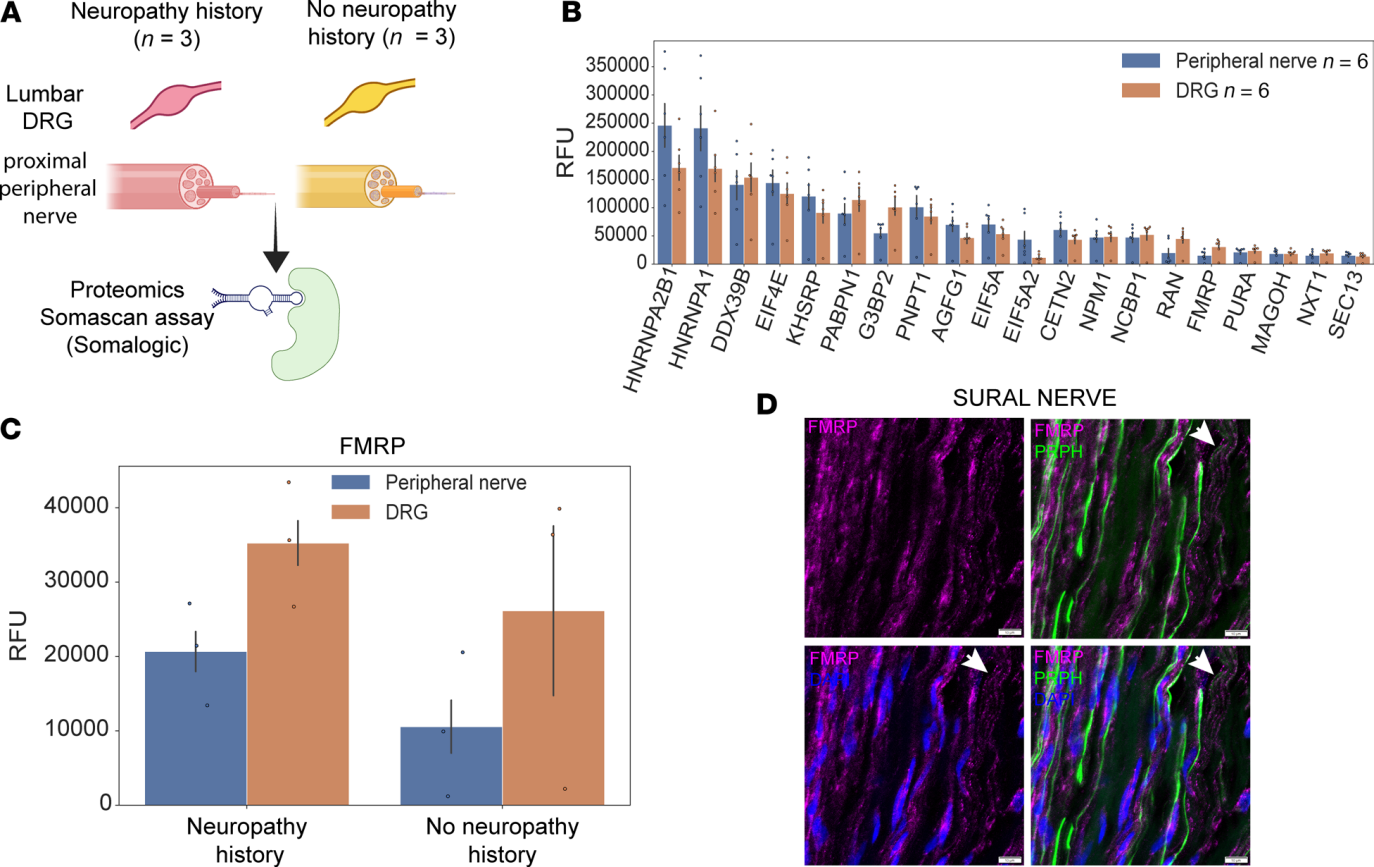
RNA-binding proteins in the human peripheral nervous system. (**A**) Overview of samples used to characterize RNA-binding proteins (RBPs). (**B**) Top RBPs associated with RNA transport in human DRG and sciatic nerve. (**C**) Changes in FMRP in peripheral nerve and DRG from donors with and without pain history. (**D**) We detect the presence of FMRP in human sural axons using IHC. Arrows point to area where FMRP is not colocalized with DAPI staining. Scale bars: 10 μm.

Along with *cis*-acting elements (or motifs) usually found in the 3′-untranslated region of mRNAs (58, 59), RBPs are required for RNA transport (60), and this process can be species specific (61–63). After demonstrating that the neuronal genes *SCN9A*, *SCN10A*, *TRPV1*, *PRPH*, and *NTRK1* are present in peripheral nerve axons, we sought to identify which RBPs were present in human peripheral nerves. We performed the SomaScan proteomic assay (https://somalogic.com) and detected 1,890 RBPs, including 40 RBPs with a role in RNA transport (based on the RBP database RBP2GO; ref. 64), in paired L4 DRG and peripheral nerves from the same organ donors (*N* = 6; Figure 7, A and B, and Supplemental Data File 12). RBPs such as fragile X mental retardation protein (FMRP), which is encoded by the *FMR1* gene, are known to play an important role in RNA transport (65, 66). We found that FMRP was robustly detected in the DRG and sciatic nerve samples from organ donors, including those with neuropathy history (Figure 7C). Using IHC, we validated that FMRP protein was present within human sural nerves, showing that the protein localizes to distal sensory axons (Figure 7D). This suggests that key RBPs involved in axonal RNA transport, including FMRP, are expressed in human sensory neurons and their peripheral axons, supporting a role for local RNA regulation in human nerve function and potentially in neuropathy states.

## Discussion

Peripheral nerves are responsible for signal transduction to and from the central nervous system (CNS). In humans, sensory axons extend for a large distance, and maintaining axonal integrity is crucial for normal function. Axonal damage such as that observed in DPN typically affects sensory axons in a length-dependent manner, highlighting the importance of uncovering the pathogenic cellular and molecular alterations, including changes in axonal mRNA transport. Previous human and rodent studies implicated that inflammatory mediators released by different cell types play an important role in the development and progression of DPN (67). Additionally, studies on sural nerve biopsies found alterations in immune response, calcium signaling, and axon guidance (68–70). In our study, we identified 2,268 genes differentially expressed between control and DPN nerves. Gene ontology analysis revealed that DPN is characterized by a coordinated pathway-level shift: genes upregulated in DPN are enriched for broad inflammatory programs and related cytokine-mediated signaling terms, whereas downregulated genes cluster in neurodevelopmental and sensory-signaling pathways, aligning with current literature. Elevated *CSF3* (encoding granulocyte colony-stimulating factor [G-CSF], a key cytokine that mobilizes and activates neutrophils), *CXCR1*, and *CXCR2* suggest enhanced neutrophil chemotaxis, consistent with recent studies reporting neutrophil involvement in DPN-associated vascular and tissue damage (71–73). Rodent data show that exogenous G-CSF modestly preserves small-fiber density, but results are inconsistent (72). Thus, elevated *CSF3* may signify both a reparative response and a driver of neutrophil-mediated damage, positioning the G-CSF/CSF3 axis as a promising yet underexplored therapeutic target in DPN. The increase in *CALCA* (encoding CGRP) and *TAC1* supports ongoing neurogenic inflammation and nociceptive signaling, in line with their known contributions to peripheral sensitization, despite limited direct data in DPN, to our knowledge. Several downregulated genes in DPN nerves suggest impaired neurovascular support, immune regulation, and axonal stability. Downregulation of *CCL14*, *PLA2G2D*, and *SIGLEC1* underscores a concerted loss of antiinflammatory and reparative immune functions in DPN nerves. *CCL14* is a human-specific CCR1/CCR5 ligand. *CCL14* normally attracts monocytes and macrophages through CCR1/CCR5 signaling, chemokine axes that support debris clearance and early neuroprotective responses after nerve injury (74, 75). Its reduced expression in human DPN nerves could therefore limit recruitment of reparative macrophages, skewing the microenvironment toward unresolved inflammation and impaired regeneration. *PLA2G2D* (encoding phospholipase A_2_ group IID) has been shown to lead to the production of pro-resolving lipid mediators (76), and *SIGLEC1* labels immunoregulatory macrophages (77); reduced expression of all these molecules suggests limited recruitment of reparative macrophages, impaired resolution of inflammation, and diminished immune regulation. Collectively, this shift may lock the nerve into a state of chronic neuroinflammation, contributing to ongoing axonal damage and hindering effective repair in DPN. Furthermore, *STMN2* (encoding stathmin-2) is a neuron-specific, microtubule-regulating protein that is crucial for axonal outgrowth, maintenance, and regeneration. *STMN2* has been implicated in peripheral neuropathies and motor denervation (78), which suggests that its decrease in DPN may contribute to axonal vulnerability. Altogether, these transcriptional changes underscore a shift toward axonal degeneration and ineffective resolution of injury in DPN, complementing the proinflammatory and fibrotic processes highlighted in our upregulated gene profile.

We also characterized human tibial and sural nerves using a multiomics approach. We found distinct pathways enriched in sural (sensory) and tibial (mixed sensory and motor) nerves following DPN. In sural nerves, we observed an enrichment in non-neuronal pathways including vasculature development and neutrophil migration. These findings support the notion that inflammatory processes and vascular alterations are pathologically important in DPN, affecting nerve function indirectly through microenvironment changes (79). In tibial nerves, the enriched pathways consisted of axonal biology–related terms. This suggests that the mechanisms of injury and repair may vary between different peripheral nerves in DPN. Patients with DPN often describe sensory symptoms such as pain, tingling, or numbness, but motor symptoms like weakness and loss of coordination are also reported, particularly in patients with distal symmetric sensorimotor polyneuropathy (80). Additionally, nerve conduction studies have demonstrated that alterations in the amplitude of motor nerve fibers generally occur after those observed in sensory nerve fibers (81). Unlike the sural nerve, which is a purely sensory nerve, the tibial nerve contains a mix of motor and sensory axons, so it is feasible that this difference contributes to transcript differences seen in our paired samples. Future studies characterizing pure motor nerves at the same distal level could also shed light on potential differences.

An important component of neuronal homeostasis is neuroimmune and neuroglial interactions (82, 83), and inflammatory mediators released by different cell types play an important role in the development and progression of DPN as previously reported (67). Schwann cells are essential for the structure and function of peripheral nerves and contribute to DPN pathogenesis via oxidative stress, endoplasmic reticulum stress, and inflammation (84–86). Additionally, their impaired ability to produce neurotrophic factors crucial for nerve health, coupled with dyslipidemia associated with diabetes that alters the composition of myelin sheaths, leads to axonal degeneration and aberrant signal transmission (84). Using our spatial RNA sequencing data, we were able to identify 8 major cell types: perineurial cells, Schwann cells, immune cells, extracellular matrix fibroblasts, adipocytes, vascular smooth muscle cells/pericytes, endothelial cells, and infiltrating immune cells. Using our cell type deconvolution approach, we found that non-myelinating Schwann cells were the most affected, possibly as consequence of unmyelinated axon loss in advanced DPN. Both myelinating and non-myelinating Schwann cells are affected by DPN. Myelinating Schwann cells are primarily responsible for forming the myelin sheath around axons, but in DPN, they often exhibit metabolic dysregulation due to the effects of hyperglycemia and disrupted insulin signaling, leading to myelin sheath abnormalities and nerve function impairment (87). Non-myelinating Schwann cells, which enwrap multiple unmyelinated axons, are also crucial for the maintenance and integrity of peripheral nerves (88); however, in DPN, these cells are more susceptible to the toxic effects of hyperglycemia, impairing their ability to support unmyelinated nerve axons that are responsible for nociception (89). Previous research has shown that non-myelinating Schwann cells create wide signaling networks with immature Schwann cells and macrophages in an attempt to protect nerve function (90).

We observed changes with axonal loss severity progression, including a decrease in Schwann cells and an increase in infiltrating immune cells, in line with previous literature (40, 91, 92). The earliest shift was a reduction in perineurial cells in nerves with moderate axonal loss compared with normal nerves, consistent with clinical and experimental evidence that blood–nerve/perineurial barrier dysfunction is crucial for the development of DPN (93). Progression from moderate to severe axonal loss was accompanied by an expansion of infiltrating immune cells and endothelial cells, paralleling single-cell and bulk studies that document mounting neuroinflammation and microvascular remodeling in diabetic nerves (94). Extracellular matrix fibroblasts became enriched only in the normal-versus-severe comparison, in line with histological and proteomic reports that fibrosis and collagen deposition are late-stage phenomena in diabetic nerves (95). Together, our results support a sequential model in which perineurial barrier breakdown sets the stage for immune and vascular infiltration, followed by fibrotic expansion and Schwann cell depletion, thereby defining discrete windows for targeted intervention at early, intermediate, and late stages of axonal loss in DPN.

Our cell-cell communication analysis revealed shared pathways and also unique pathways between moderate and severe axonal loss. In a sample with moderate axonal loss, a pro-healing signature persists, including the presence of SEMA7A, ANGPT2, and VEGF signaling pathways. These signals are known to support axon guidance, angiogenesis, and nerve repair (96–98). In contrast, TGF-β1 and tenascin/syndecan signaling pathways emerged only in nerves with severe axonal loss, indicating a switch to a profibrotic, non-regenerative state. These findings align with reports that TGF-β1 drives Schwann cell–mediated fibrosis in advanced nerve damage states (99, 100). After peripheral nerve injury, TGF-β1 rises transiently, promoting Schwann cell dedifferentiation and migration that facilitate axonal regrowth (101). Persistent activation, however, directs Schwann cells and fibroblasts toward a collagen-rich, fibrotic phenotype that impedes remyelination (102, 103). A comparable, chronically elevated TGF-β1 signature in DPN fosters the same fibrotic, proinflammatory milieu, further compromising nerve function (104). Tenascin C binding to syndecan-4 promotes fibroblast matrix contraction and chronic tissue remodeling (105). Together, their concerted activation marks a transition beyond which functional repair is likely restricted in advanced DPN; however, it also delineates potential therapeutic targets for early, targeted intervention. We also observed that the top ligands expressed in the nerves can interact with receptors in DRG neurons. Interestingly, ligands such as *APP* and *C3* are involved in axonal reorganization and remodeling, which suggests that peripheral nerve cells can release mediators that directly affect axons. Complement C3 has been associated with increased risk of neuropathy (106), suggesting that it may have a proinflammatory role that limits the nerve regeneration capacity. Similarly, APP has been associated with neuronal death in the CNS (107, 108) and may contribute to axonal degeneration in DPN.

Because of shifts in peripheral nerve cell types identified between DPN sural nerves with moderate and severe axonal loss using our spatial approach, we set out to investigate the molecular changes using bulk RNA sequencing. Peripherin is an intermediate neurofilament and a structural component of axons. It has been identified as a marker of axonal damage (54, 109) and functions in neurite stability and axonal transport (110). Peripherin antibodies were present in patients with type 1 diabetes, and a reduction in peripherin expression was observed to accompany hyperalgesia in a rat streptozotocin-induced type 1 diabetes model (110, 111). In our study, we observed an increase of peripherin mRNA in DPN sural nerves with severe axonal loss, suggesting that *PRPH* can also be a marker of axonal loss in human sural nerves. A previous study found that diabetic mice lacking neurofilaments experienced conduction velocity slowing and decreased nerve action potential amplitude, unlike those with normal neurofilaments, which showed only mild neuropathy, irrespective of hyperglycemia levels (112). This indicates that neurofilaments help axons resist damage caused by diabetes. We also observed that caveolin-1 (*Cav1*) is upregulated in DPN sural nerves with severe axonal loss. Previous studies in mice had shown that absence of Cav1 correlated with increased DPN severity, including motor and sensory nerve conduction velocities, and mechanical or thermal sensitivity (113) and that low levels of Cav1 contribute to demyelination (114). Cav1 has also been associated with pain development (115) as well as anti-inflammation and neuroprotection (116). These observations are in line with our cell-cell interaction analysis of Visium samples and suggest that there are regenerative pathways present in peripheral nerves that can be targeted to facilitate recovery in DPN.

In this study, we also characterized the expression of neuronal mRNAs *SCN9A*, *SCN10A*, *TRPV1*, *NTRK1*, and *PRPH* with RNAscope in situ hybridization in human sural and sciatic nerves. We demonstrated axonal localization of these mRNAs, consistent with our RNA sequencing data. We qualitatively observed similar levels of each mRNA in both sciatic nerves isolated from organ donors and sural nerves from surgeries. Future studies using fiber type–specific markers, including those for sympathetic and sensory nerves, may help identify the specific nerve fiber populations involved. Axonal mRNA transport is important for development, regeneration, and response to injury or endogenous molecule signaling (117). During pathfinding in normal development, axons respond to neurotropic cues through local protein synthesis (118). After tissue damage, translation can be initiated in DRG neurons and their axons, resulting in sensory neuron sensitization (119). Local mRNA translation can be advantageous by allowing for faster responses to stimuli than axoplasmic transport of proteins, which can take hours or days to traffic proteins to peripheral axon segments (120). Following axonal translation, locally synthesized mRNAs can also be retrogradely transported to neuronal soma and alter nuclear transcription, raising another mechanism through which peripheral protein synthesis can mediate nociceptive response (31, 66, 121). While rodent sensory axon transport and local protein synthesis had been shown for some mRNAs relevant to nociception, including Na_v_1.8 (35, 119), there is still much work to be done to characterize which mRNAs are translated downstream of nociceptive input, particularly in human peripheral nerves. In this study, we have identified mRNAs that are localized to human sensory axons. Revealing which RNAs are transported into axons, and therefore can potentially be translated locally, could result in additional therapeutic targets for pain and axonal regeneration in DPN and other peripheral neuropathies.

Directing mRNAs to specific subcellular sites requires 3 major components: (a) *cis*-acting elements within the mRNA, most frequently found in the 3′-untranslated region; (b) RBPs that can recognize and bind to the *cis*-acting elements in a sequence-specific manner; and (c) the resulting ribonucleoprotein complex that can, then, be linked directly to motor proteins or hitchhike to vesicles such as lysosomes and mitochondria for transport to a specific subcellular region (122). Our analysis revealed that several RBPs are present in peripheral nerves, including fragile X mental retardation protein (FMRP). FMRP is a regulatory RBP that controls mRNA transport and local translation with known roles in neurodevelopment and synapse function (65, 66). Previous studies have shown that disruption of RBPs involved in mRNA axonal transport can consequently lead to the loss of axonal integrity (123). This can result in neuronal degeneration and cause neurological diseases such as fragile X syndrome (124), amyotrophic lateral sclerosis (125), spinal muscular atrophy (126), and peripheral neuropathy (127). Additionally, peripheral neuropathy is a common feature of fragile X–associated tremor/ataxia syndrome (FXTAS), involving damage to the peripheral nerve (128–130). FXTAS is a neurodegenerative disorder in individuals with the fragile X mental retardation 1 (FMR1) premutation that leads to cognitive impairment, tremors, and neuropathy. Males carrying the *FMR1* premutation showed a loss of distal reflexes and a reduction in vibratory perception, and a strong correlation was identified between CGG repeat length and total neuropathy score in both males and females (130). The X-inactivation ratio, which determines the relative expression of normal versus premutation alleles, is thought to influence the prevalence and severity of symptoms in females (131). In line with these studies, our proteomics data show the potential involvement of FMRP in DPN, with this RBP being present in human DRG and peripheral nerves with a history of neuropathy.

Our study has several limitations: (a) apart from a subset of DPN sural nerves, our peripheral nerve specimens are not clinically characterized; (b) some comparisons involve small sample sizes owing to the limited availability of human nerve tissue and the high cost of advanced spatial-omics techniques; and (c) our mRNA colocalization studies with nerve fiber markers are qualitative, and we did not perform quantitative analysis of axonal mRNA puncta owing to limitations of quantifying the axonal space in the imaging frame. Future studies using larger sample sizes and leveraging higher-resolution techniques, such as imaging-based spatial transcriptomics, are needed to address these constraints.

Overall, our study provides fundamental insight into human peripheral nerves and DPN. With increase in axonal loss, we observe a competitive interplay between regenerative and degenerative processes in DPN. On the one hand, there is an increase in immune cells and extracellular matrix as well as proinflammatory mediators that contribute to an inflammatory and degenerative phenotype. On the other hand, we detect mediators that can be targeted to activate regenerative pathways. Early targeting of genes and pathways involved in regenerative processes can open therapeutic avenues that directly treat DPN in peripheral nerves. Future studies evaluating the functional effects of specific cell types and mediators will be key to achieving this goal. In addition, we identified the presence of specific mRNAs in the axons of human peripheral nerves, supporting our hypothesis that RNA transport occurs in human sensory nerves. It is likely that this is an important mechanism for the maintenance of axonal integrity and peripheral nerve homeostasis.

## Methods

Further information can be found in Supplemental Methods.

### Sex as a biological variable.

The demographics of our DPN population are 75% male (Figure 1, Supplemental Data File 1, and Supplemental Tables 1–3), limiting our analysis of sex differences and interpretation of results. However, to account for potential differences due to sex, we included sex as a factor in our design formula for DESeq2 analysis. Additionally, for the control-versus-DPN comparison, samples were age- and sex-matched.

### Statistics: differential expression analysis of bulk RNA sequencing datasets.

We performed differential expression analysis using the DESeq2 package (132) (based on the negative binominal distribution and Wald statistics). Nominal *P* values were corrected for multiple testing using the Benjamini-Hochberg method (85). In addition, we performed shrinkage of the log_2_ fold change (LFC), using the adaptive shrinkage estimator from the “ashr” R package (86), and set the contrast to “sural” versus “tibial” or “moderate” versus “severe” as the groups we wanted to compare. Genes were differentially expressed (DE) if FC was greater than 1.5 and adjusted *P* value was less than 0.05. For differential expression analysis between control and DPN sural nerve samples, we set the contrast to “DPN” versus “control” as the groups we wanted to compare; genes were considered DE if FC was greater than 2 and adjusted *P* value was less than 0.001. Statistical hypothesis testing results for all tests can be found in Supplemental Data Files 3, 7, and 10. For each gene tested, we report baseMean (mean of normalized counts), LFC, lfcSE (standard error of the LFC estimate), *P* value (Wald test *P* value), and Padj (adjusted *P* values).

### Statistics: Visium spatial transcriptomic integration and cell type annotation.

We used R (v4.4.2) and Seurat (v5.3.0) (133) for data analysis. We used Seurat’s spatially resolved RNA sequencing data analysis workflow and used H5 files as input for the spatial analysis. We applied the Seurat layer integration workflow to analyze 6 spatial transcriptomics datasets from human sural nerves (*n* = 5) representing normal, moderate, and severe axonal loss. Individual samples were processed, annotated with metadata, and normalized with sctransform (134). Datasets were merged and filtered by unique reads (500–15,000) and gene (>200) counts per spot to exclude low-quality data. Harmony (IntegrateLayers), (135) principal component analysis, and uniform manifold approximation and projection (UMAP) were used for batch correction and dimensionality reduction. Clustering was done with FindNeighbors and FindClusters (133), and cell types were annotated using canonical markers. For spot deconvolution, we used STdeconvolve (48), a reference-free deconvolution tool. Cell types were identified based the top markers in each topic.

### Statistics: Visium cell type proportion testing.

The proportion of enriched cell groups was computed used the package DittoSeq (136), and enriched cell type proportion differences between groups were assessed using scProportionTest with permutation-based testing (137).

### Statistics: Visium cell-cell interactions.

We used the CellChat (v2.1.2) spatial transcriptomics workflow to identify cell-cell interactions between cell types enriched in moderate and severe axonal loss samples (49). Separate CellChat objects were generated for each sample, incorporating normalized RNA data from the single-cell transform assay, cell type annotations, and spatial coordinates, to construct spatially informed CellChat objects for downstream communication analysis. Following the standard CellChat pipeline, we identified overexpressed genes and ligand-receptor interactions. Communication probabilities were calculated using computeCommunProb() with the truncated mean method (type = “truncatedMean,” trim = 0.1). To incorporate spatial proximity, we set interaction.range = 250 μm to restrict overall signaling to biologically plausible distances and enabled contact.dependent = TRUE with contact.range = 100 μm to limit contact-dependent signaling to closely adjacent cells. The communication network was refined using filterCommunication(), excluding interactions with fewer than 10 cells. To identify robust signaling patterns, we compared the 3 severe samples with the moderate case (S8) by calculating pairwise differences (S11–S8, S12–S8, S14–S8). Pathways consistently up- or downregulated across all comparisons were retained, and top pathways were ranked by average absolute change. Unique pathways in S8 and severe samples were also noted. Key pathways were visualized using CellChat bubble plots and heatmaps.

### Study approval.

This work was approved by the University of Texas at Dallas Institutional Review Board (IRB-24-380, tibial and sural nerves from amputation surgeries; IRB-24-120, control sural nerves from cross-facial nerve graft surgeries; and IRB-24-326, DRG and peripheral nerves from organ donors).

### Data and code availability.

RNA sequencing data are available in the Database of Genotypes and Phenotypes (dbGaP; phs001158). The processed data can be accessed at the following link: https://sensoryomics.shinyapps.io/human-peripheral-nerves/ Code is available at https://github.com/orgs/utdal/teams/utdpaincenter/ Values for all data points in graphs are reported in the Supporting Data Values file.

## Author contributions

DTF, TL, DKW, and TJP designed the study. DTF performed RNA extractions for bulk RNA sequencing. DTF, SS, and IS performed Visium experiments. DTF, SS, AS, and BQS performed RNAscope and IHC experiments. JMM performed SomaScan assay and analysis. DTF analyzed sequencing data. MK, NNI, and KBM assisted in the analysis of bulk RNA sequencing. EEU performed sural nerve morphology and qualitatively determined axonal density. TL, DKW, and GLT collected tibial and sural nerve samples. GLT coordinated clinical aspects of the study. SMR collected control sural nerves. DTF, BQS, and TJP wrote the manuscript. All authors read and edited the paper.

## Supplementary Material

Supplemental data

Supplemental data set 1

Supplemental data set 2

Supplemental data set 3

Supplemental data set 4

Supplemental data set 5

Supplemental data set 6

Supplemental data set 7

Supplemental data set 8

Supplemental data set 9

Supplemental data set 10

Supplemental data set 11

Supplemental data set 12

Supporting data values

## Version 1 08/19/2025

In-Press Preview

## Version 2 10/15/2025

Electronic publication
