# Supplementary material for: Cell and molecular profiles in peripheral nerves shift toward inflammatory phenotypes in diabetic peripheral neuropathy
Source: J Clin Invest. 2025 Aug 19;135(20):e184075. doi: 10.1172/JCI184075 (PMC12520680; doi:10.1172/JCI184075)
Supplement: Supplemental data [file jci-135-184075-s170.pdf]

SUPPLEMENTARY MATERIALS

Supplementary Methods:

Consent, tissue and patient data collection. All protocols were reviewed and approved by the UT Dallas (UTD) and UT Southwestern Medical Center (UTSWMC) Institutional Review Boards. Patients undergoing lower leg amputation at two major tertiary care hospitals (Clements University Hospital (CUH) at UTSWMC and Parkland Memorial Hospital (PMH)) in Dallas, Texas were recruited as part of the study. Informed consent for participation was obtained for each patient during study enrolment. Tibial and sural nerves were recovered from diabetic patients who have non-reconstructable soft tissue or bone loss, recalcitrant bone infection (osteomyelitis) and/or critical limb ischemia and had been advised to undergo lower extremity amputation. The tibial and sural nerves were harvested from the part of the leg which was amputated (distal specimen). The specimens were placed in sterile specimen cups, snap-frozen in liquid nitrogen and stored in a – 80C freezer. Non-DPN sural nerves were recovered from non-diabetic patients having lower leg amputation due to trauma or non-reconstructable deformity. Details can be found in **Suppl. File 1**. Additional controls (peripheral nerves) were recovered from organ donors at Southwest Transplant Association within 4 hours of cross-clamp, frozen immediately on crushed dry ice, and stored in a -80°C freezer as previously described (1) and non-DPN sural nerves obtained from cross facial nerve grafts in facial paralysis surgeries at UTSW. Sural nerves were harvested through a small 3 cm vertical incision midway between the lateral malleolus and Achilles tendon. Once an adequate length was confirmed for cross facial nerve grafting, the nerve was transected proximally and distally using tenotomy scissors. The remaining portion not used in the cross facial nerve grafting was frozen on pulverized dry ice, then transferred to a sterile vial and stored at –80°C. (**Suppl. Table S1**). All human tissue procurement procedures from organ donors were also approved by the Institutional Review Boards at the University of Texas at Dallas.

Michigan Neuropathy Screening Instrument. The Michigan Neuropathy Screening Instrument (MNSI) was administered to 6 patients undergoing lower leg amputation as previously described (2, 3) and included 15 yes/no questions assessing foot symptoms such as pain, numbness, and temperature sensitivity, along with one item each for general weakness and peripheral vascular symptoms. This was followed by a brief clinical exam assessing foot appearance (deformities, skin changes, ulcers), vibration sensation at the great toe (normal, reduced, or absent), and ankle reflexes (normal, reduced, or absent). Foot deformities included hammer toes, hallux valgus, and Charcot foot. Higher total scores reflected greater neuropathy severity.

Tissue preparation. For Visium, Xenium, RNAscope and/or immunohistochemistry (IHC), tissues were gradually embedded in optimal cutting temperature (OCT) compound in a cryomold by adding small volumes of OCT over dry ice to avoid thawing. Nerves used for Visium and Xenium were cryosectioned onto Visium slides at 10μm. Tissues used for RNAscope and IHC were sectioned onto SuperFrost Plus charged slides at 20μm. Xenium protocol was performed at K2-Biolabs according to the manufacturer's instructions.

Peripheral nerve morphology. Frozen sural nerve biopsies received on dry ice were rapidly thawed for 1 minute at 37°C in a water bath, immediately fixed by immersion in 3% glutaraldehyde in 0.1 M phosphate buffer at room temperature for 12-15 hours, post-fixed in 1% osmium tetroxide for 2 hours, embedded in Epoxy resin, sectioned at 1 μm and stained with 1% Toluidine Blue (in 2% sodium borate in distilled water), as previously published (4, 5). Semi-qualitative assessment of axonal density/loss was determined by a board-certified clinical neuromuscular pathologist, based on accepted age- adjusted convention as follows: 0-10% loss = normal; 11-30% loss = mild; 31-69% = moderate; >70% = severe.

RNAscope in situ hybridization. RNAscope in situ hybridization multiplex version 2 was performed as instructed by Advanced Cell Diagnostics (ACD) and as previously described (6).

Optimal results were observed with protease digestion time of 10 seconds. **Suppl. Tables S4** and **S5** contain information on patients/donor tissues used and probes. All tissues were checked for RNA quality by using a positive control probe cocktail (ACD) which contains probes for high, medium and low-expressing mRNAs that are present in all cells (ubiquitin C > Peptidyl-prolyl cis-trans isomerase B > DNA-directed RNA polymerase II subunit RPB1). A negative control probe against the bacterial DapB gene (ACD) was used to reference non-specific/background label.

Immunohistochemistry (IHC). For dual RNAscope/IHC, after completion of RNAscope in situ hybridization, slides were incubated in blocking buffer (10% Normal Goat Serum, 0.3% Triton-X 100 in 0.1M PB) for 1 hour at room temperature while being shielded from light. Slides were placed in a light-protected humidity-controlled tray and incubated in primary antibody (Chicken Polyclonal Antibody to Peripherin, dilution 1:500, Encor Biotechnology, catalog number CPCA-Peri; Mouse Monoclonal Antibody to SOX10, dilution 1:40, Abcam, catalog number ab216020; goat polyclonal antibody to SOX10, dilution , Novus Biologicals/R&D Biotechne, catalog number AF2864; mouse monoclonal anti-beta-tubulin III (TUJ1), dilution 1:100, BioLegend, catalog number 801202) in blocking buffer for 3 hours at room temperature or overnight at 4°C. Slides were washed with 0.1 M PB, then incubated in secondary antibody for 1 hour at room temperature. Slides were washed with 0.1 M PB, air-dried, and cover slipped with Prolong Gold Antifade mounting medium. For regular IHC, slides were kept in the -20 C cryostat chamber for 15 minutes following completion of sectioning. The slides were then immediately fixed in ice-cold formalin (10%) for 1 minute followed by dehydration in 50% ethanol (1 minute), 70% ethanol (1 minute), and 100% ethanol (2 minutes) at room temperature. The slides were briefly air dried. A hydrophobic pen (ImmEdge PAP Pen; Vector Labs) was used to draw boundaries around each tissue section, and boundaries were allowed to air dry. Slides were incubated with blocking buffer (10% Normal Goat Serum, Atlanta Biologicals, Cat

#S13150h, 0.3% Triton X-100 in 0.1 M PB) for 1 hour at room temperature. Sections were then incubated overnight with a primary antibody cocktail. Following primary antibody incubation, sections were washed with 0.1 M phosphate buffer and incubated with Alexa Fluor secondary antibodies (Fisher Scientific, dilutions 1:1000) for 1 hour at room temperature. Sections were washed in 0.1 M phosphate buffer. To remove lipofuscin signal, Trublack (1:20 in 70% ethanol; Biotium #23007) was pipetted to cover each section for 1 minute before being rinsed off. Finally, slides were air dried and cover slipped with Prolong Gold Antifade reagent (Fisher Scientific; P36930).

Image acquisition. Sciatic and sural nerve sections were imaged on an Olympus FV3000 confocal microscope at 100X magnification. A minimum of 2 images were acquired for each nerve section. The area imaged was randomly chosen; however, we prioritized sections that did not have any sectioning artifact and sections that contained intact peripherin/Beta-tubulin III fiber staining. The acquisition parameters were set based on guidelines for the FV3000 provided by Olympus. Briefly, the gain was kept at the default setting 1, HV ≤ 600, offset was based on HI-LO settings, and laser power ≤ 5%.

RNA-seq library preparation.

Bulk RNA-sequencing

Following RNA purification, cDNA libraries were prepared with TruSeq Stranded Total RNA Library Prep with ribosomal RNA depletion for all samples according to the manufacturer’s instructions (Illumina). The quality of the extracted RNA and cDNA at each library preparation step was assessed with Qubit (Invitrogen) and High Sensitivity NGS fragment analysis kit on the Fragment Analyzer (Agilent Technologies). The amount of cDNA was standardized across samples and the libraries were sequenced on Illumina NextSeq500 sequencing machines with 75-bp single-end reads. mRNA library preparation and sequencing were done at the Genome Center in the University of Texas at Dallas Research Core Facilities. For control versus DPN

samples, libraries were sequenced on Illumina NovSeq X sequencing machine with 150-bp paired-end reads (Psomagen).

Visium Spatial Gene Expression

Visium tissue optimization and spatial gene expression protocols were followed exactly as described by 10x Genomics (https://www.10xgenomics.com/) using Haematoxylin and Eosin as the counterstain. Optimal permeabilization time was obtained at 12 min incubation with permeabilization enzyme. Imaging was conducted on an Olympus vs120 slide scanner. Sural nerves from 5 patients were used. mRNA library preparation and sequencing were done at the Genome Center in the University of Texas at Dallas Research Core Facilities.

RNA-seq – mapping raw counts and alignment of barcoded spots with imaged sections.

Bulk. Sequenced reads were trimmed to avoid compositional bias and lower sequencing quality at either end and to ensure all quantified libraries were mapped with the same read length, and mapped to the GENCODE reference transcriptome (v27) in a strand-aware and splicing-aware fashion using the STAR alignment tool (7). Stringtie (8) was used to generate relative abundances in Transcripts per Million (TPM), and non-mitochondrial coding gene abundances were extracted and renormalized to a million to generate coding TPMs for each sample.

Visium. The output data of each sequencing run (Illumina BCL files) was processed using the Space Ranger (v1.1) pipelines provided by 10x Genomics. Samples were demultiplexed into FASTQ files using Space Ranger's mkfastq pipeline. Space Ranger's count pipeline was used to align FASTQ files with brightfield microscope images previously acquired, detect barcode/UMI counting, and map reads to the human reference transcriptome (Gencode v27 and GRCh38.p10) (9). This pipeline generates, for each sample, feature-barcode matrices that contain raw counts and places barcoded spots in spatial context on the slide image (cloupe

files). Gene expression with spatial context can, then, be visualized by loading cloupe files onto Loupe Browser (v5, 10x Genomics).

Somascan Assay

Tissue lysates were prepared from fresh-frozen DRGs and peripheral nerves. The tissues were placed in T-PER Tissue Protein Extraction Reagent (Thermo Scientific, Cat # 78510) with additional 1X Halt Protease Inhibitor Cocktail (Thermo Scientific, Cat # 87786) and homogenized using Precellys Soft Tissue Homogenizing beads (Bertin Corp, Cat # P000933-LYSK0-A.0). Samples were centrifuged at 14,000 x g for 15 minutes in the cold room. The resulting supernatant was quantified Micro BCA™ Protein Assay Kit (Thermo Scientific, Cat# 23235) and normalized accordingly. Proteins were profiled using the SOMAScan platform. 7000 analytes were measured on the SOMAScan assay. Quality controls were performed by SomaLogic to correct for technical variabilities within-run and between-run for each sample.

Supplementary figures:

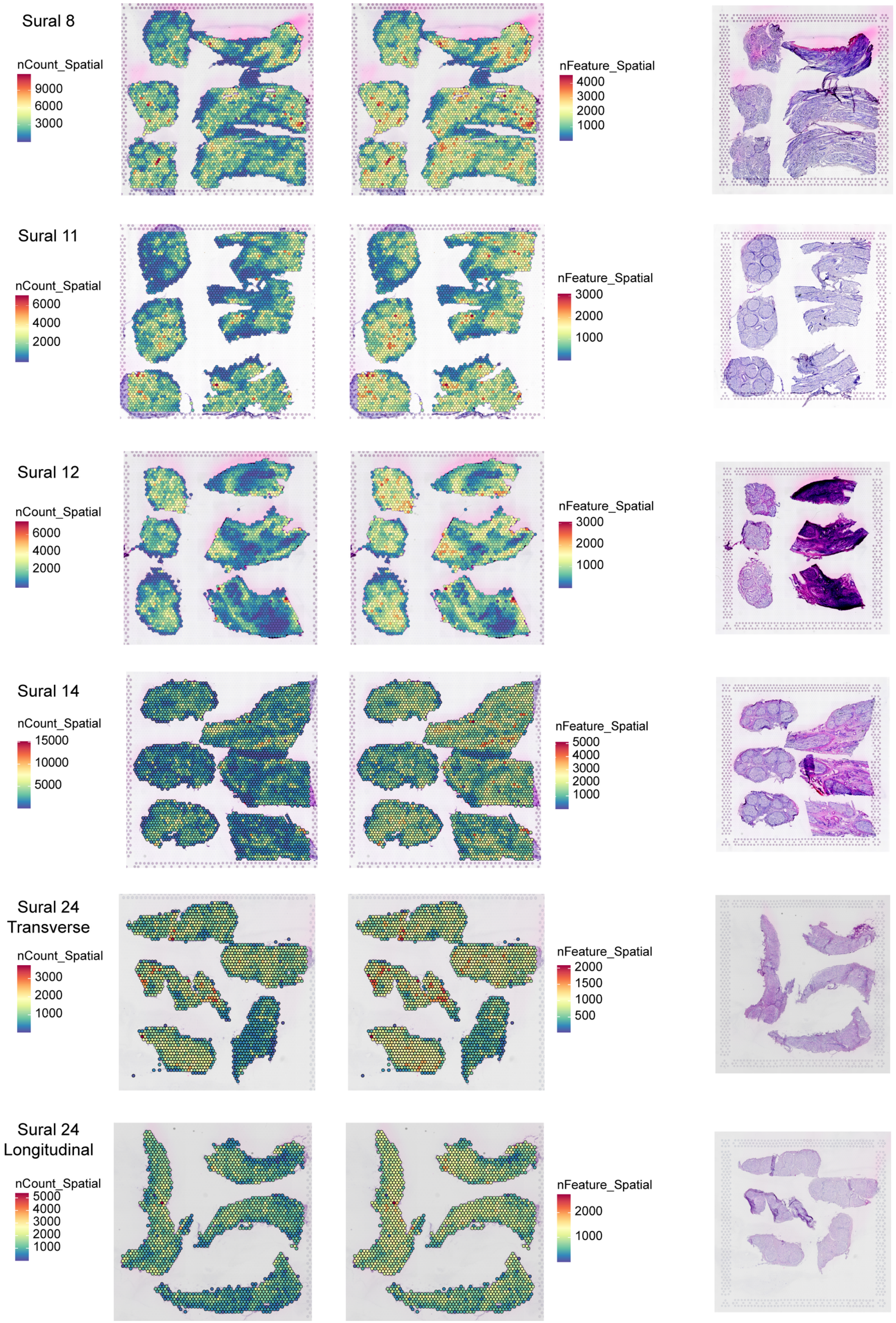
Sural 8
nCount_Spatial
9000
6000
3000
nFeature_Spatial
4000
3000
2000
1000
Sural 11
nCount_Spatial
6000
4000
2000
nFeature_Spatial
3000
2000
1000
Sural 12
nCount_Spatial
6000
4000
2000
nFeature_Spatial
3000
2000
1000
Sural 14
nCount_Spatial
15000
10000
5000
nFeature_Spatial
5000
4000
3000
2000
1000
Sural 24
Transverse
nCount_Spatial
3000
2000
1000
nFeature_Spatial
2000
1500
1000
500
Sural 24
Longitudinal
nCount_Spatial
5000
4000
3000
2000
1000
nFeature_Spatial
2000
1000

Suppl. Figure 1. Quality control of sural nerves. Plots display number of reads (nCount_Spatial) and number of genes (nFeature_Spatial) next to respective H&E image for each sural nerve.

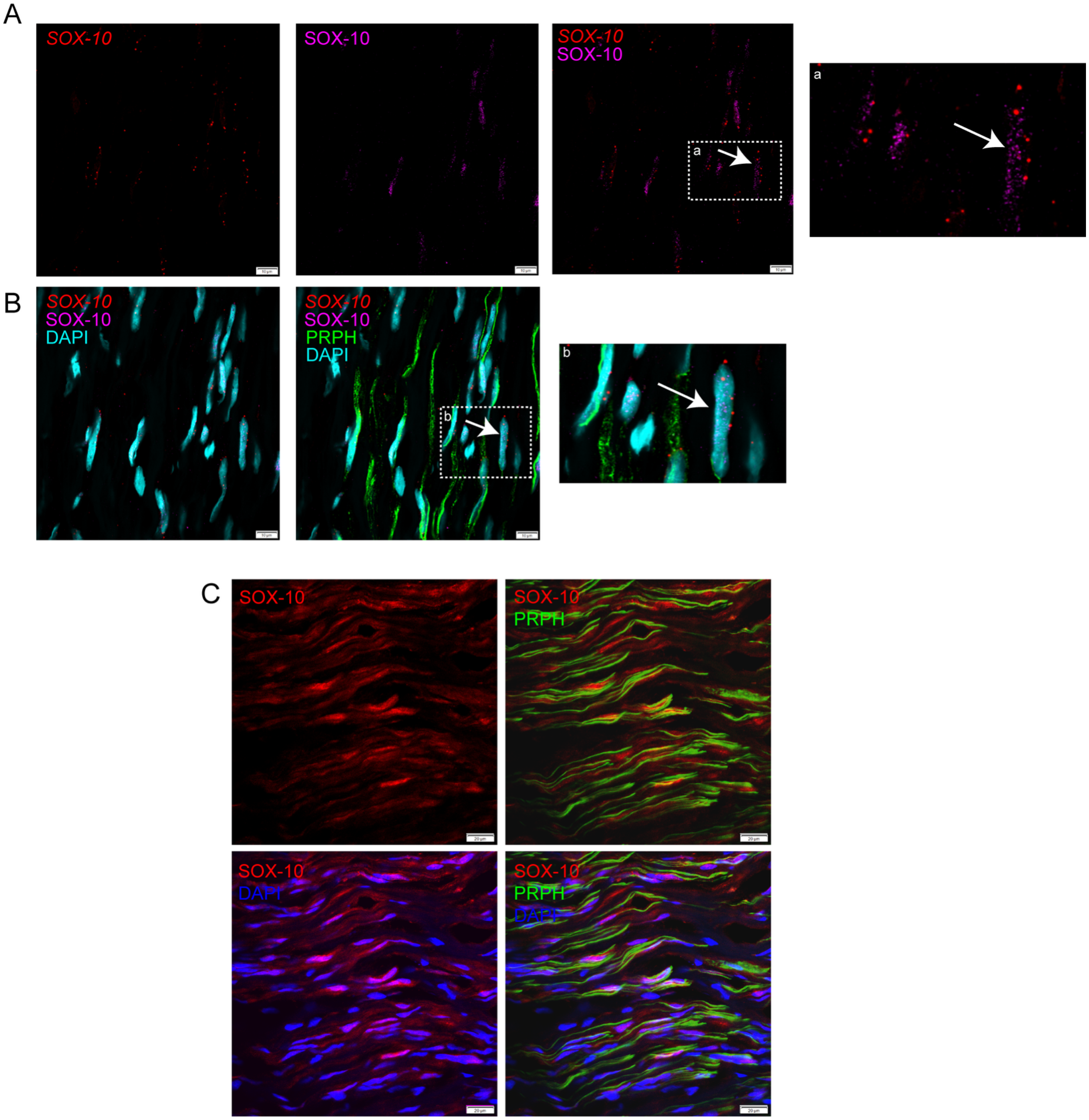

Suppl. Figure 2. SOX10+ Schwann cells are present in human peripheral nerves. **A**) *SOX10* mRNA puncta (red) colocalize with SOX10 protein (magenta). **B**) *SOX10* mRNA puncta (red) colocalize with DAPI (cyan). Peripherin (PRPH, green) was used to label nerve fibers.

Insets show zoomed-in images. Scale bars=10 µm. C) Expression of SOX10 protein using a second antibody. Scale bars=10 µm.

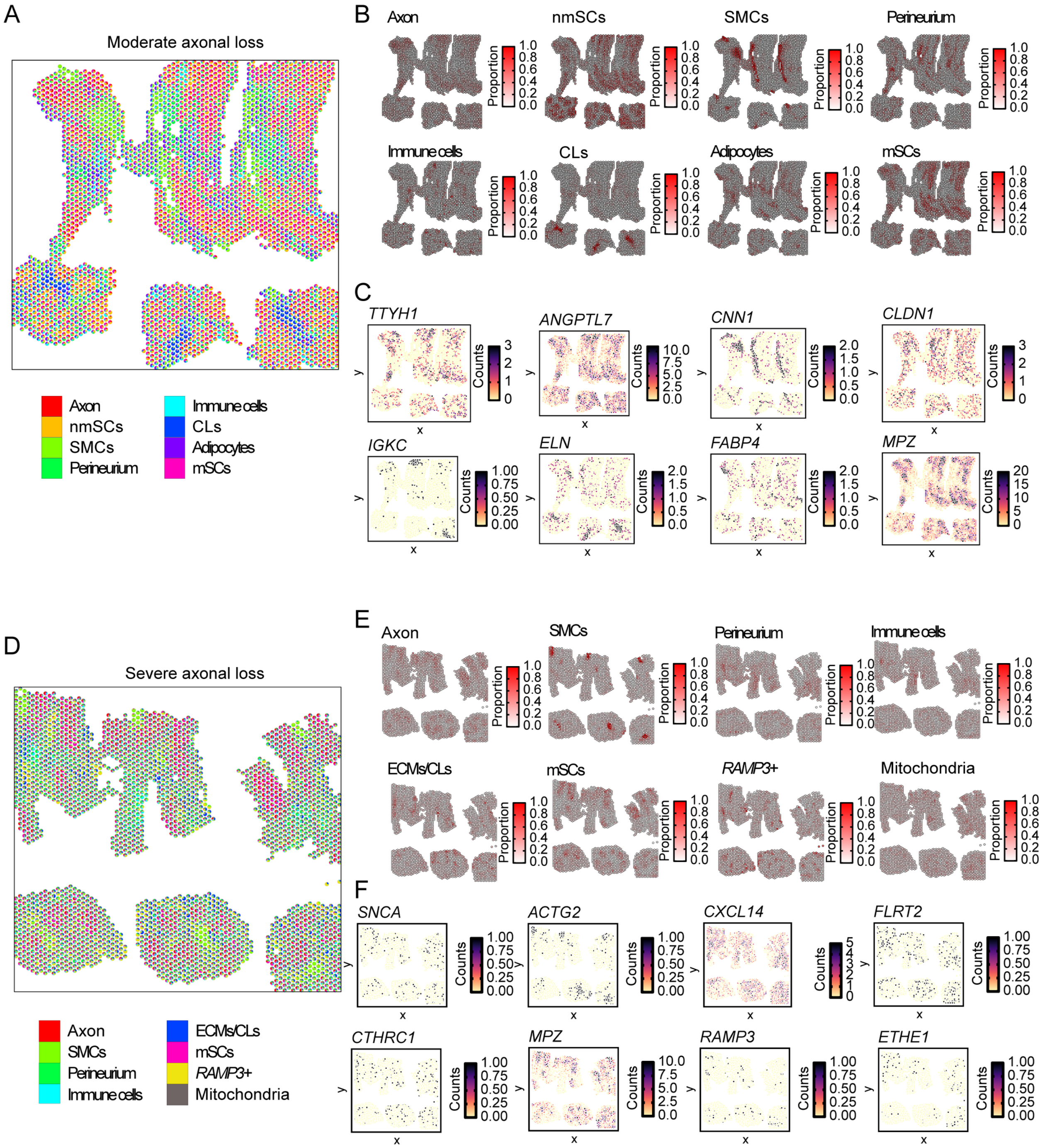

Suppl. Figure 3. Cell type deconvolution of moderate and severe axonal loss sural nerves. Deconvolution of cells in a sample with moderate axonal loss **(A)** and in a sample with severe axonal loss **(D)**. Proportion of cells identified in the sample with moderate axonal loss **(B)** and in the sample with severe axonal loss **(E)**. Markers for each cell type in sample with moderate axonal loss **(C)** and with severe axonal loss **(F)**. SMCs=Smooth muscle cells;

ECM=Extracellular matrix; CLs= Connective layers; nmSCs= non-myelinating Schwann cells; mSCs= myelinating Schwann cells.

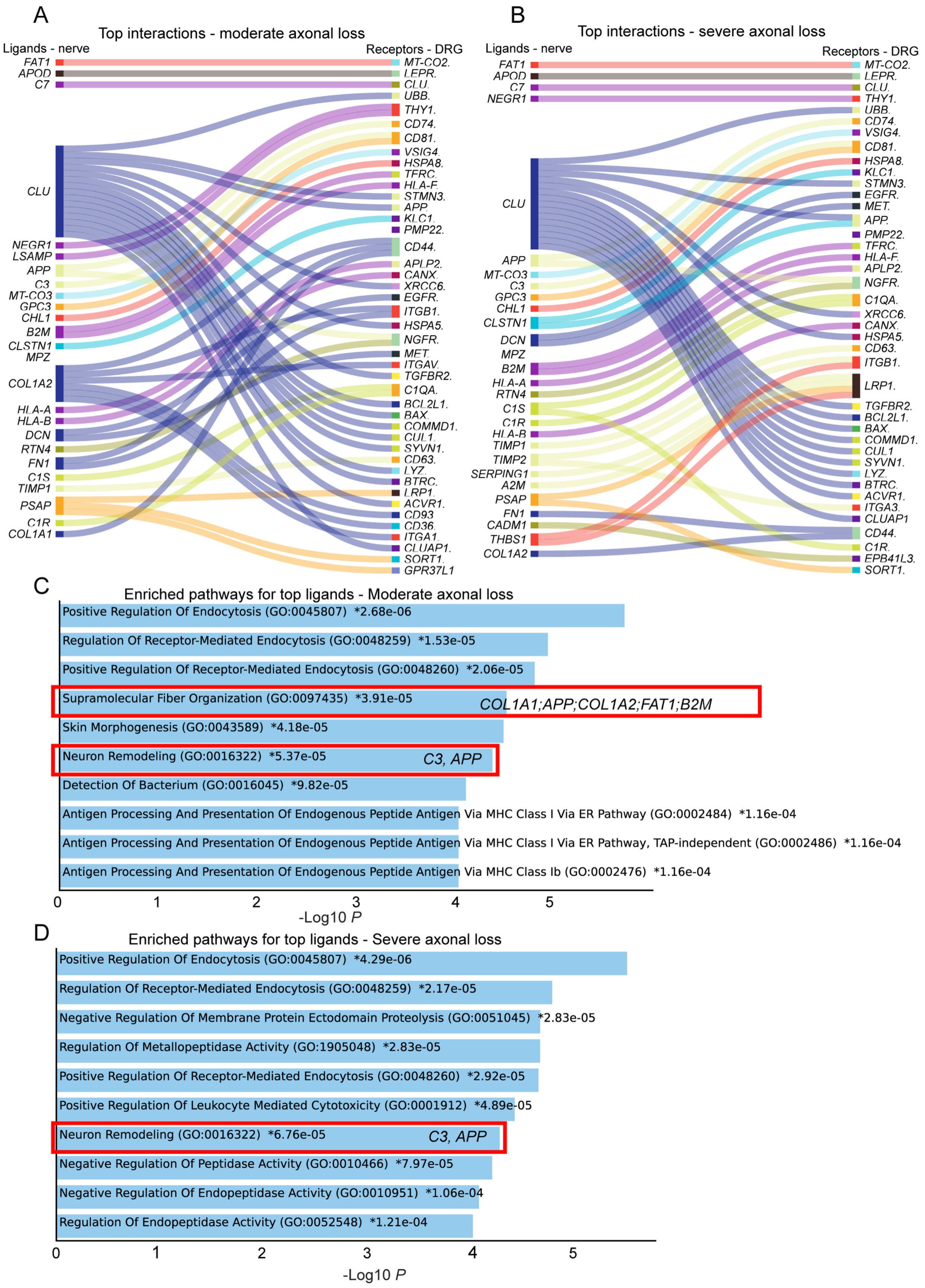
A
Top interactions - moderate axonal loss
Ligands - nerve
Receptors - DRG
FAT1
APOD
C7
CLU
NEGR1
LSAMP
APP
C3
MT-CO3
GPC3
CHL1
B2M
CLSTN1
MPZ
COL1A2
HLA-A
HLA-B
DCN
RTN4
FN1
C1S
TIMP1
PSAP
C1R
COL1A1
MT-CO2.
LEPR.
CLU.
UBB.
THY1.
CD74.
CD81.
VSIG4.
HSPA8.
TFRC.
HLA-F.
STMN3.
APP.
KLC1.
PMP22.
CD44.
APLP2.
CANX.
XRCC6.
EGFR.
ITGB1.
HSPA5.
NGFR.
MET.
ITGAV.
TGFBR2.
C1QA.
BCL2L1.
BAX.
COMMD1.
CUL1.
SYVN1.
CD63.
LYZ.
BTRC.
LRP1.
ACVR1.
CD93.
CD36.
ITGA1.
CLUAP1.
SORT1.
GPR37L1
B
Top interactions - severe axonal loss
Ligands - nerve
Receptors - DRG
FAT1
APOD
C7
NEGR1
CLU
APP
MT-CO3
C3
GPC3
CHL1
CLSTN1
DCN
MPZ
B2M
HLA-A
RTN4
C1S
C1R
HLA-B
TIMP1
TIMP2
SERPING1
A2M
PSAP
FN1
CADM1
THBS1
COL1A2
MT-CO2.
LEPR.
CLU.
THY1.
UBB.
CD74.
VSIG4.
CD81.
HSPA8.
KLC1.
STMN3.
EGFR.
MET.
APP.
PMP22.
TFRC.
HLA-F.
APLP2.
NGFR.
C1QA.
XRCC6.
CANX.
HSPA5.
CD63.
ITGB1.
LRP1.
TGFBR2.
BCL2L1.
BAX.
COMMD1.
CUL1
SYVN1.
LYZ.
BTRC.
ACVR1.
ITGA3.
CLUAP1
CD44.
C1R.
EPB41L3.
SORT1.
C
Enriched pathways for top ligands - Moderate axonal loss
Positive Regulation Of Endocytosis (GO:0045807) *2.68e-06
Regulation Of Receptor-Mediated Endocytosis (GO:0048259) *1.53e-05
Positive Regulation Of Receptor-Mediated Endocytosis (GO:0048260) *2.06e-05
Supramolecular Fiber Organization (GO:0097435) *3.91e-05
COL1A1;APP;COL1A2;FAT1;B2M
Skin Morphogenesis (GO:0043589) *4.18e-05
Neuron Remodeling (GO:0016322) *5.37e-05
C3, APP
Detection Of Bacterium (GO:0016045) *9.82e-05
Antigen Processing And Presentation Of Endogenous Peptide Antigen Via MHC Class I Via ER Pathway (GO:0002484) *1.16e-04
Antigen Processing And Presentation Of Endogenous Peptide Antigen Via MHC Class I Via ER Pathway, TAP-independent (GO:0002486) *1.16e-04
Antigen Processing And Presentation Of Endogenous Peptide Antigen Via MHC Class Ib (GO:0002476) *1.16e-04
0
1
2
3
4
5
-Log10 P
D
Enriched pathways for top ligands - Severe axonal loss
Positive Regulation Of Endocytosis (GO:0045807) *4.29e-06
Regulation Of Receptor-Mediated Endocytosis (GO:0048259) *2.17e-05
Negative Regulation Of Membrane Protein Ectodomain Proteolysis (GO:0051045) *2.83e-05
Regulation Of Metallopeptidase Activity (GO:1905048) *2.83e-05
Positive Regulation Of Receptor-Mediated Endocytosis (GO:0048260) *2.92e-05
Positive Regulation Of Leukocyte Mediated Cytotoxicity (GO:0001912) *4.89e-05
Neuron Remodeling (GO:0016322) *6.76e-05
C3, APP
Negative Regulation Of Peptidase Activity (GO:0010466) *7.97e-05
Negative Regulation Of Endopeptidase Activity (GO:0010951) *1.06e-04
Regulation Of Endopeptidase Activity (GO:0052548) *1.21e-04
0
1
2
3
4
5
-Log10 P

Suppl. Figure 4. Interactome analysis between ligands enriched in the nerve and DRG receptors. A) Interactome plot showing interactions between top ligands expressed in a sample with moderate axonal loss. **B)** Interactome plot showing interactions between top ligands expressed in a sample with severe axonal loss. **C, D)** Enriched pathways for top ligands in moderate **(C)** and severe **(D)** axonal loss.

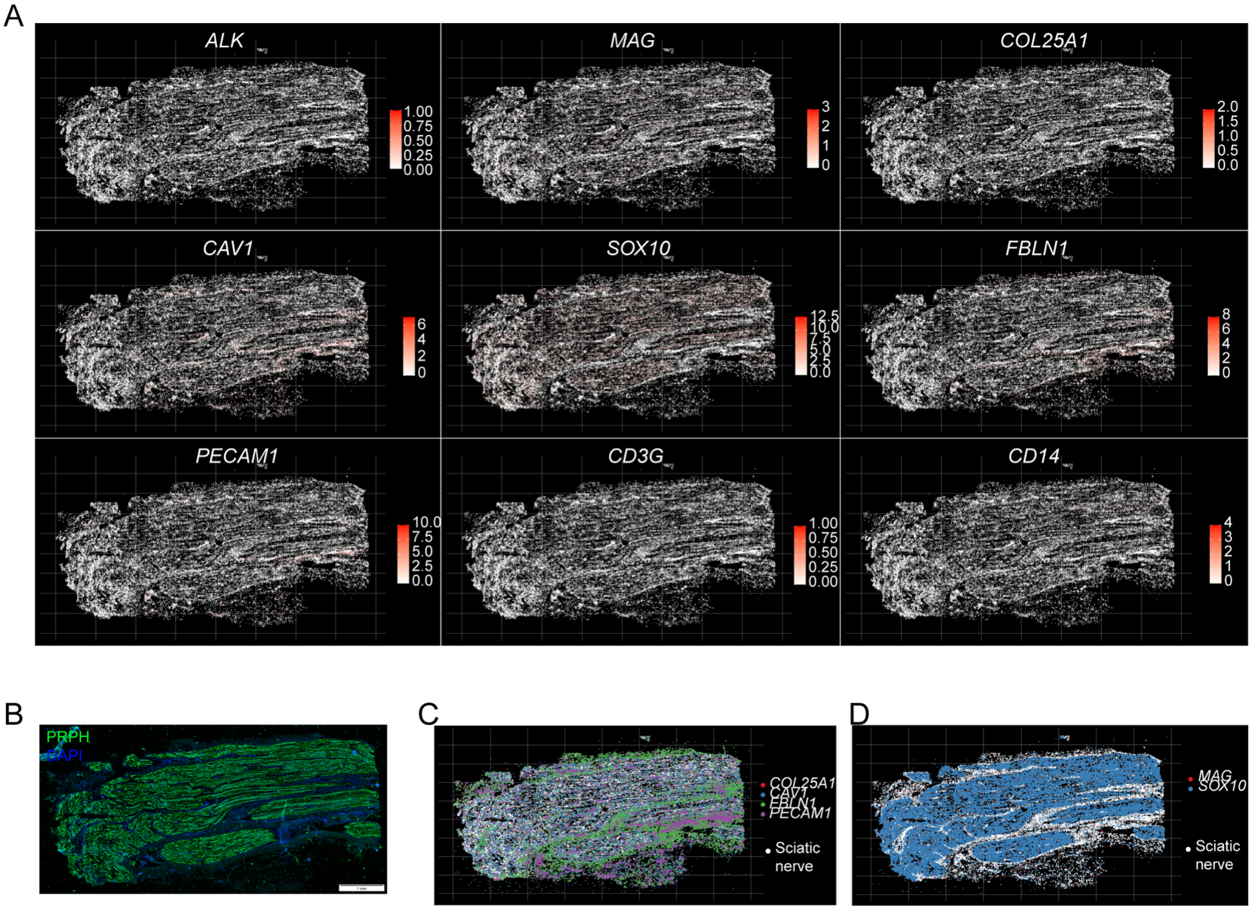

Suppl. Figure 5. Visualization of specific genes in human peripheral nerves using Xenium. A) Spatial localization of genes differentially expressed between moderate and severe axonal loss: ALK receptor tyrosine kinase (*ALK*), myelin associated glycoprotein (*MAG*), calveolin-1 (*CAV1*) and Collagen Type XXV Alpha 1 Chain (*COL25A1*) and cell type markers (SOX10-Schwann cells, PECAM1-endothelial cells, FBLN1-fibroblasts, CD3G-T cells, CD14-macrophages). B) Immunohistochemistry staining showing nerve fibers labeled with PRPH (green) and DAPI (blue). C) Co-localization of *CAV1* and *COL25A1* with fibroblast and endothelial cell markers. D) Co-localization of *MAG* with Schwann cell marker.

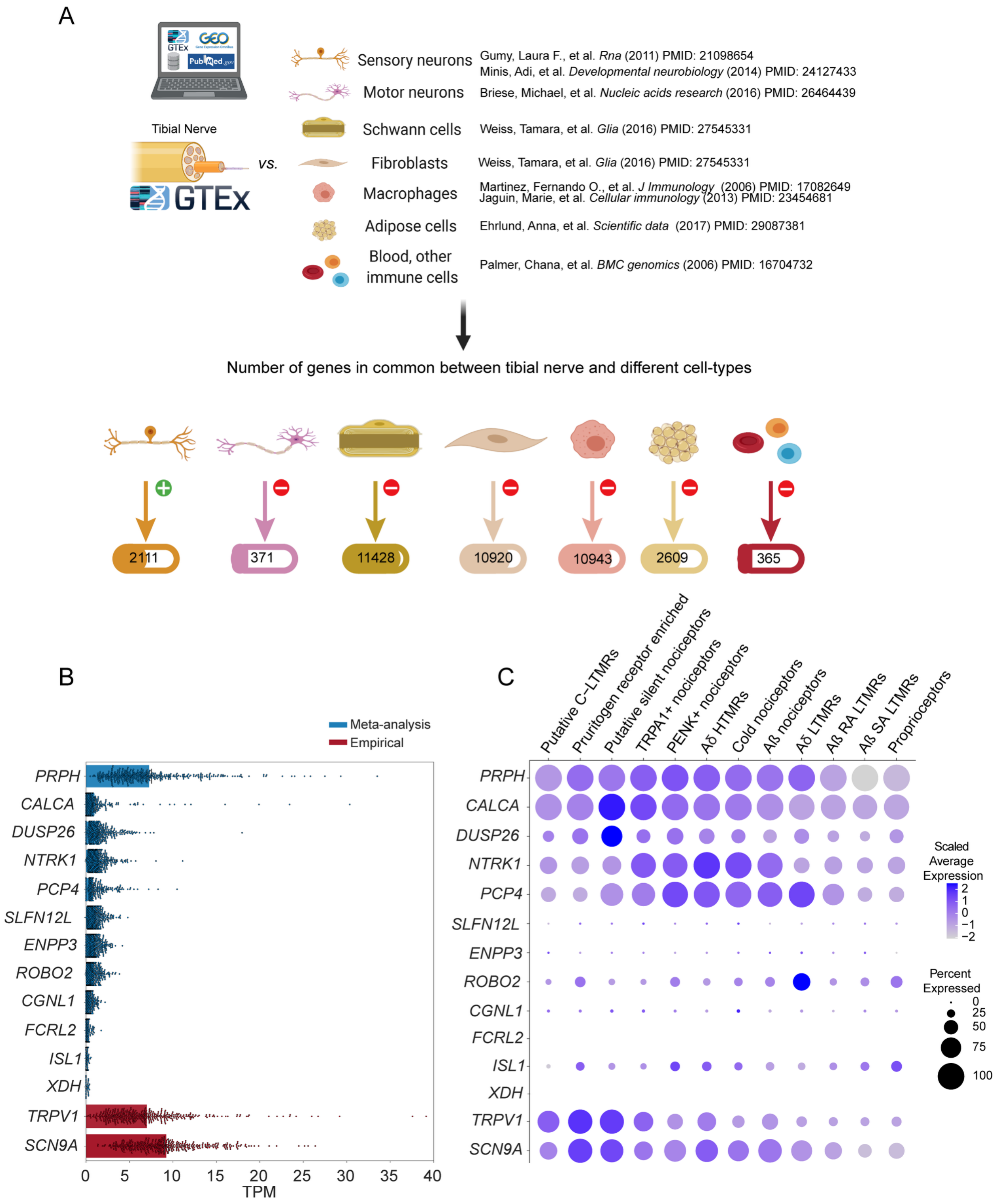

Suppl. Figure 6. Meta-analysis of putative axonal mRNAs. A) Diagram of publicly available studies included in meta-analysis. **B)** List of genes that were present only in sensory axons and that are likely axonal mRNAs (blue). In red are genes that are not exclusively axonal. **C)** Dot plot showing how putative axonal genes are expressed in human dorsal root ganglia (DRG) neurons. The size of the dot represents the percentage of barcodes within a cluster, and the

color corresponds to the average expression (scaled data) across all barcodes within a cluster for each gene shown.

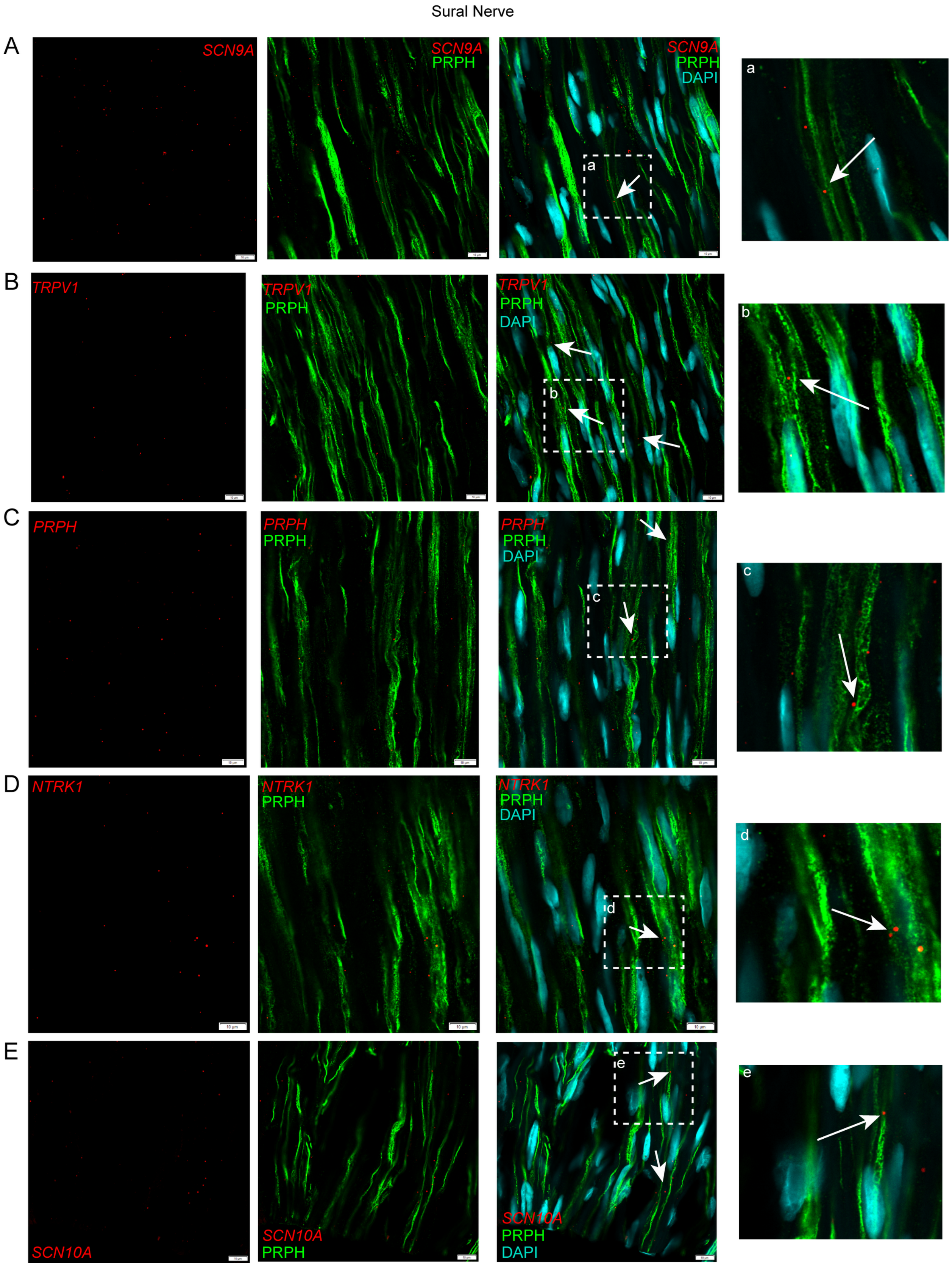

Sural Nerve
A
SCN9A
SCN9A
PRPH
SCN9A
PRPH
DAPI
a
a
B
TRPV1
TRPV1
PRPH
TRPV1
PRPH
DAPI
b
b
C
PRPH
PRPH
PRPH
PRPH
PRPH
DAPI
c
c
D
NTRK1
NTRK1
PRPH
NTRK1
PRPH
DAPI
d
d
E
SCN10A
SCN10A
PRPH
SCN10A
PRPH
DAPI
e
e

Suppl. Figure 7. Specific mRNA expression in sural nerves using RNAscope and IHC. A-E) mRNA puncta for *SCN9A* (A), *TRPV1* (B), *PRPH* (C), *NTRK1* (D) and SCN10A (E) are shown in red and colocalize with peripherin (PRPH, green), a general marker of nerve fibers, in sural nerves. Arrows indicate regions where mRNA puncta do not overlap with DAPI (cyan), suggesting axonal-specific localization. Insets show magnified views. Scale bars = 10 μm.

Sciatic Nerve

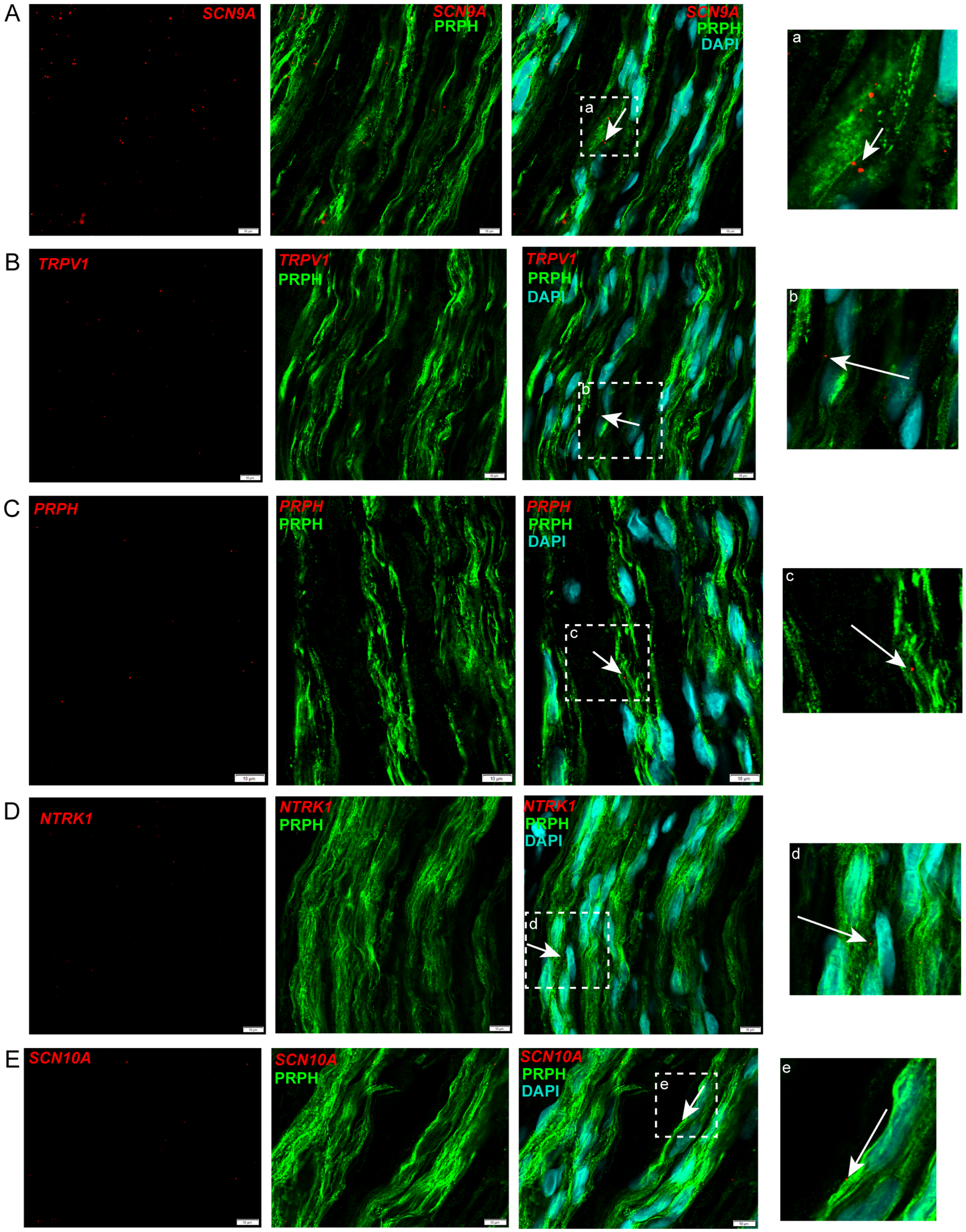

Suppl. Figure 8. Specific mRNA expression in sciatic nerves using RNAscope and IHC. A-E) mRNA puncta for *SCN9A* (A), *TRPV1* (B), *PRPH* (C), *NTRK1* (D) and SCN10A (E) are shown in red and colocalize with peripherin (PRPH, green), a general marker of nerve fibers, in sural nerves. Arrows indicate regions where mRNA puncta do not overlap with DAPI (cyan), suggesting axonal-specific localization. Insets show magnified views. Scale bars = 10 µm.

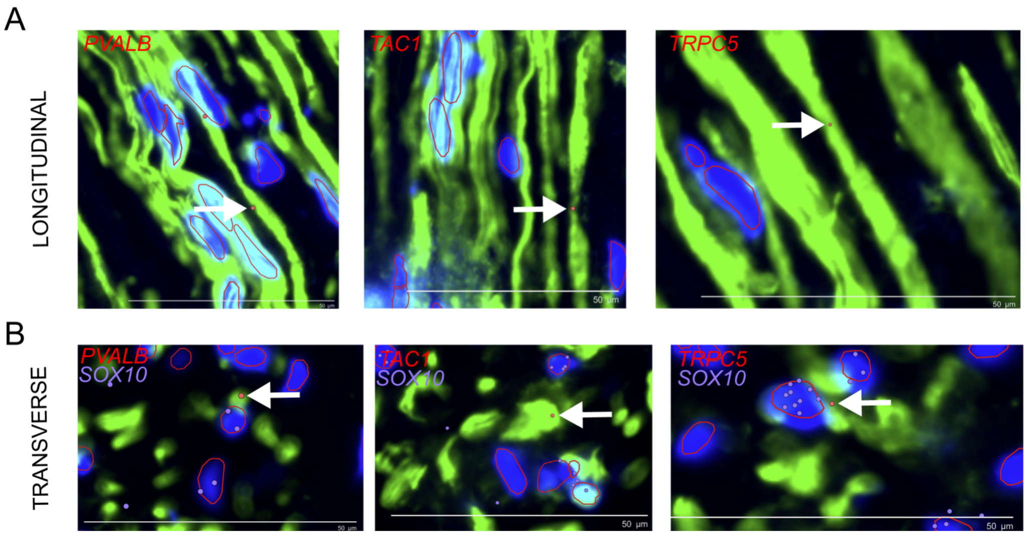

Suppl. Figure 9. Axonal mRNAs are detected using Xenium in situ. We used the off-the-shelf Xenium Brain panel and detect the presence of neuronal markers parvalbumin (*PVALB*), tachykinin precursor 1 (*TAC1*) and transient receptor potential cation channel subfamily c member 5 (*TRPC5*) in human peripheral axons (labeled by peripherin in green, PRPH) in longitudinal **(A)** and transverse **(B)** sections. SRY-Box Transcription Factor 10 (*SOX10*) is a marker of Schwann cells and its expression in DAPI+ cells surrounding the nerve fibers was used to assist in the validation of our cell segmentation. Scale bar= 50 µm.

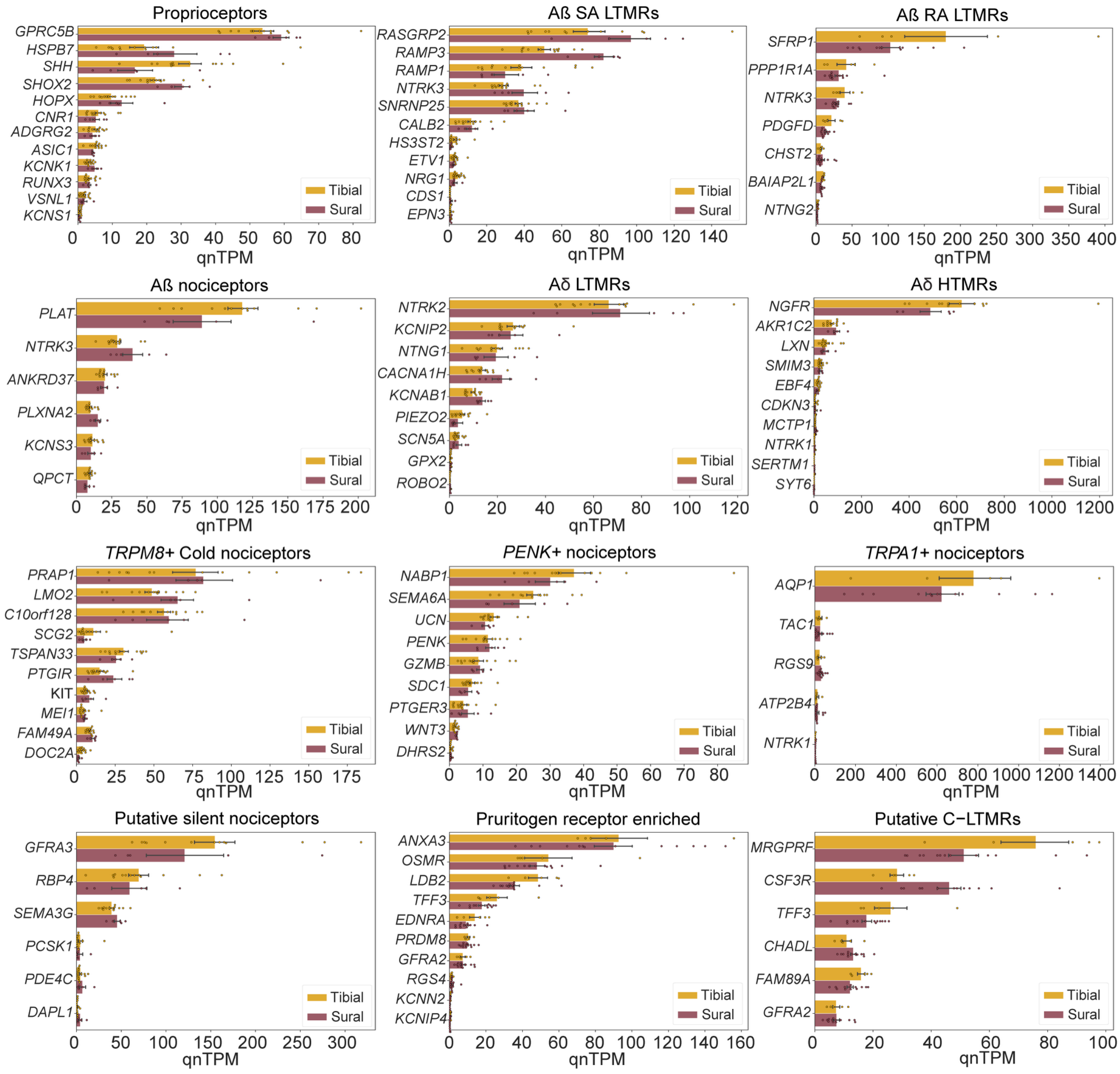

Suppl. Figure 10. Markers of different neuronal subtypes can be identified in human sural and tibial nerves.

Suppl. Tables:

Table S1: Patient Information for DPN versus control sural nerve bulk RNA-seq analysis.

Subject	Age	Sex	Diabetes?	DPN	MSNI	Pain score	Pain description	DPN related medication	Related medical history
**C1**	36	Male	No	No	-	-	-	-	-
**C2**	73	Male	No	No	-	-	-	-	-
**C3**	52	Female	No	No	-	-	-	-	-
**C4**	40	Male	No	No	-	-	-	-	-
**C5**	59	Female	No	No	-	-	-	-	-
**C6**	60	Male	No	No	-	-	-	-	-
**S38**	31	Male	Type II	Yes	8	7	Throbbing Pain	Tramadol	left charcot foot w/ chronic osteomyelitis
**S39**	79	Male	Type II	Yes	7	5	Sharp Pain	Hydrocodone	osteomyelitis, right ankle and foot
**S40**	53	Female	Type II	Yes	8	-	Numbness	Gabapentin (in the past for burning pain)	osteomyelitis, left ankle
**S42**	51	Male	Type II	Yes	8	-	Numbness	-	Acute hematogenous osteomylitis, unspecified site
**S43**	59	Female	Type II	Yes	9	-	Burning Pain (Overbearing)	Mentions "pain medication"	Diabetic neurogenic arthropathy
**S44**	60	Male	Type II	Yes	8	4	Dull Pain	Mentions "pain medication"	Non healing left heel wound; PVD disease; Diabetic infection of left foot; Gangrene; Acute blood loss anemia; CAD, multiple vessel; Septic Shock; PAD

Table S2. Patient Information for sural versus tibial nerve bulk RNA-seq analysis.

Subject	Age	Sex	Diabetes
**S8**	75	M	type II
**S11**	80	M	type II
**S12**	61	M	type II
**S13**	61	F	type II
**S14**	50	M	type II

Table S3. Patient Information for moderate versus severe axonal loss bulk RNA-seq analysis

Subject	Age	Sex	Diabetes	Axonal density/loss
**S8**	75	M	type II	Moderate Axonal Loss
**S10**	62	F	type II	Moderate Axonal Loss
**S11**	80	M	type II	Severe Axonal Loss
**S12**	61	M	type II	Severe Axonal Loss
**S13**	61	F	type II	Severe Axonal Loss
**S14**	50	M	type II	Severe Axonal Loss
**S18**	52	M	type II	Severe Axonal Loss
**S19**	66	M	type II	Severe Axonal Loss
**S20**	57	M	type II	Moderate Axonal Loss
**S21**	39	M	type II	Severe Axonal Loss
**S22**	63	F	type II	Severe Axonal Loss
**S25**	65	F	type II	Severe Axonal Loss
**S28**	47	F	type II	Severe Axonal Loss
**S30**	52	M	type II	Severe Axonal Loss
**S31**	57	M	type II	Severe Axonal Loss
**S33**	70	M	type II	Moderate Axonal Loss
**S34**	50	M	type II	Severe Axonal Loss

Table S4: Tissue Donor/Patient Information for RNAscope/IHC.

Donor/Patient	Tissue	Sex	Age	Cause of Death	Diabetes	Axonal loss
D1	Peripheral nerve	M	37	Anoxia/overdose	No	-
D2	Peripheral nerve	F	38	Anoxia/Asphyxiation/Natural Causes	No	-
P1	Sural nerve	M	52	Not Applicable	Type II	Severe
P2	Sural nerve	M	60	Not Applicable	No	Mild
P3	Sural nerve	F	62	Not Applicable	Type II	Moderate
P4	Sural nerve	M	61	Not Applicable	Type II	Severe
P5	Sural nerve	M	50	Not Applicable	Type II	Severe
P6	Sural nerve	F	63	Not Applicable	Type II	Severe
P7	Sural nerve	M	70	Not Applicable	Type II	Moderate

Table S5: RNAscope probes.

Probe	Gene Name	ACD Probe Catalog Number
NTRK1	Neurotrophic Receptor Tyrosine Kinase 1	402631
PRPH	Peripherin	410231
SCN9A	Sodium Voltage-Gated Channel Alpha Subunit 9; Nav1.7	562251
SCN10A	Sodium Voltage-Gated Channel Alpha Subunit 10; Nav1.8	406291
SOX10	SRY-Box Transcription Factor 10	484121
TRPV1	Transient Receptor Potential Cation Channel Subfamily V Member 1	415381

Supplemental Data Files 1–12:

Suppl. File 1. (separate Excel file) Patient information. Details on age, sex, diabetes, surgery procedure, related medical history, specimen collected, nerve morphology, sequencing and RNAscope.

Suppl. File 2. (separate Excel file) TPM values for control and DPN sequencing data.

Suppl. File 3. (separate Excel file) Results of statistical analysis for DPN versus control samples.

Suppl. File 4. (separate Excel file) Gene enrichment analysis for genes downregulated in DPN nerves.

Suppl. File 5. (separate Excel file) Gene enrichment analysis for genes upregulated in DPN nerves.

Suppl. File 6. (separate Excel file) TPM values for tibial and sural sequencing data.

Suppl. File 7. (separate Excel file) Results of statistical analysis for sural versus tibial.

Suppl. File 8. (separate Excel file) Gene enrichment analysis for genes upregulated in sural nerves.

Suppl. File 9. (separate Excel file) Gene enrichment analysis for genes upregulated in tibial nerves.

Suppl. File 10. (separate Excel file) Results of statistical analysis for severe versus moderate axonal loss sural nerves.

Suppl. File 11. (separate Excel file) Gene enrichment analysis for genes differential expressed between severe versus moderate axonal loss sural nerves.

Suppl. File 12. (separate Excel file) Proteomics data. RNA-binding proteins (RBPs) detected in human DRG and sciatic nerves using Somascan assay.

Suppl. References:

1. Shiers SI, Sankaranarayanan I, Jeevakumar V, Cervantes A, Reese JC, and Price TJ. Convergence of peptidergic and non-peptidergic protein markers in the human dorsal root ganglion and spinal dorsal horn. *J Comp Neurol.* 2021;529(10):2771-88.
2. Feldman EL, Stevens MJ, Thomas PK, Brown MB, Canal N, and Greene DA. A practical two-step quantitative clinical and electrophysiological assessment for the diagnosis and staging of diabetic neuropathy. *Diabetes Care.* 1994;17(11):1281-9.
3. Herman WH, Pop-Busui R, Braffett BH, Martin CL, Cleary PA, Albers JW, et al. Use of the Michigan Neuropathy Screening Instrument as a measure of distal symmetrical peripheral neuropathy in Type 1 diabetes: results from the Diabetes Control and Complications Trial/Epidemiology of Diabetes Interventions and Complications. *Diabet Med.* 2012;29(7):937-44.
4. Ubogu EE, Yosef N, Xia RH, and Sheikh KA. Behavioral, electrophysiological, and histopathological characterization of a severe murine chronic demyelinating polyneuritis model. *J Peripher Nerv Syst.* 2012;17(1):53-61.
5. Xia RH, Yosef N, and Ubogu EE. Clinical, electrophysiological and pathologic correlations in a severe murine experimental autoimmune neuritis model of Guillain-Barré syndrome. *J Neuroimmunol.* 2010;219(1-2):54-63.
6. Shiers S, Klein RM, and Price TJ. Quantitative differences in neuronal subpopulations between mouse and human dorsal root ganglia demonstrated with RNAscope in situ hybridization. *Pain.* 2020;161(10):2410-24.
7. Dobin A, Davis CA, Schlesinger F, Drenkow J, Zaleski C, Jha S, et al. STAR: ultrafast universal RNA-seq aligner. *Bioinformatics.* 2013;29(1):15-21.

8. Pertea M, Pertea GM, Antonescu CM, Chang T-C, Mendell JT, and Salzberg SL. StringTie enables improved reconstruction of a transcriptome from RNA-seq reads. *Nature biotechnology.* 2015;33(3):290-5.
9. Frankish A, Diekhans M, Ferreira A-M, Johnson R, Jungreis I, Loveland J, et al. GENCODE reference annotation for the human and mouse genomes. *Nucleic acids research.* 2019;47(D1):D766-D73.
